# Ensemble and single-particle level fluorescent fine-tuning of carbon dots via positional changes of amines toward “supervised” oral microbiome sensing

**DOI:** 10.1117/1.JBO.28.8.082807

**Published:** 2023-07-06

**Authors:** Fatemeh Ostadhossein, Parikshit Moitra, Maha Alafeef, Dinabandhu Sar, Shannon D’Souza, Lily F. Benig, Michael Nelappana, Xuedong Huang, Julio Soares, Kai Zhang, Dipanjan Pan

**Affiliations:** a University of Illinois at Urbana-Champaign, Department of Bioengineering, Urbana, Illinois, United States; b Carle Foundation Hospital, Mills Breast Cancer Institute, Urbana, Illinois, United States; c University of Illinois at Urbana-Champaign, Beckman Institute of Advanced Science and Technology, Urbana, Illinois, United States; d The Pennsylvania State University, Department of Nuclear Engineering, State College, Pennsylvania, United States; e Fudan University, Department of Chemistry, Shanghai, China; f University of Illinois at Urbana‐Champaign, Frederick Seitz Materials Research Laboratory, Urbana, Illinois, United States; g University of Illinois at Urbana-Champaign, School of Molecular and Cellular Biology, Department of Biochemistry, Urbana, Illinois, United States; h The Pennsylvania State University, Department of Materials Science and Engineering, University Park, Pennsylvania, United States; i The Materials Research Institute, Millennium Science Complex, University Park, Pennsylvania, United States; j Huck Institutes of the Life Sciences, University Park, Pennsylvania, United States

**Keywords:** carbon dots, fluorescence, positional isomers, single-particle imaging, dental biofilm, machine learning, bacterial sensing

## Abstract

**Significance:**

Carbon dots (CDs) have attracted a host of research interest in recent years mainly due to their unique photoluminescence (PL) properties that make them applicable in various biomedical areas, such as imaging and image-guided therapy. However, the real mechanism underneath the PL is a subject of wide controversy and can be investigated from various angles.

**Aim:**

Our work investigates the effect of the isomeric nitrogen position as the precursor in the synthesis of CDs by shedding light on their photophysical properties on the single particles and ensemble level.

**Approach:**

To this end, we adopted five isomers of diaminopyridine (DAP) and urea as the precursors and obtained CDs during a hydrothermal process. The various photophysical properties were further investigated in depth by mass spectroscopy. CD molecular frontier orbital analyses aided us in justifying the fluorescence emission profile on the bulk level as well as the charge transfer processes. As a result of the varying fluorescent responses, we indicate that these particles can be utilized for machine learning (ML)-driven sensitive detection of oral microbiota. The sensing results were further supported by density functional theoretical calculations and docking studies.

**Results:**

The generating isomers have a significant effect on the overall photophysical properties at the bulk/ensembled level. On the single-particle level, although some of the photophysical properties such as average intensity remained the same, the overall differences in brightness, photo-blinking frequency, and bleaching time between the five samples were conceived. The various photophysical properties could be explained based on the different chromophores formed during the synthesis. Overall, an array of CDs was demonstrated herein to achieve ∼100% separation efficacy in segregating a mixed oral microbiome culture in a rapid (<0.5  h), high-throughput manner with superior accuracy.

**Conclusions:**

We have indicated that the PL properties of CDs can be regulated by the precursors’ isomeric position of nitrogen. We emancipated this difference in a rapid method relying on ML algorithms to segregate the dental bacterial species as biosensors.

## 1 Introduction

As an emerging class of carbon nanomaterials, carbon dots (CDs) have garnered researchers’ interests in the past decade due to their excellent biocompatibility, replete surface functional groups, water dispersibility, and unique photoluminescence.^1^^–^^5^ These extraordinary properties have opened new avenues for their advanced application in cell labeling, bioimaging, drug delivery, sensors, and energy related devices.^6^^–^^13^

Among their favorable properties, the photoluminescence of CD has been widely utilized. CDs exhibit relatively symmetrical emission spectra on the wavelength scale, and the emission peaks are wide with a Stokes shift comparable to organic dyes.^14^^–^^17^ Interestingly, the emission spectra can be tuned by exciting the CDs at various wavelengths, a phenomenon known as wavelength dependent behavior, which can potentially be exploited for multiplexing.^18^^,^^19^ However, the real mechanism underneath the photoluminescence (PL) property is a subject of wide controversy.^20^^–^^22^

To design new carbon materials with improved properties, understanding the underlying photophysics at the molecular level is crucial; however, due to its complexity, many aspects of the mechanism remain unknown. CDs show differential photoluminescence depending on the chemical structures including graphitic conjugated cores, molecular fluorophores, and surface defect states.^23^^–^^30^ All CDs share the same ${\mathrm{sp}}^{2}$ graphitic core, which may contain nitrogen as part of an aromatic ring, despite their different synthesis routes. It has also been proposed that CD surfaces contain moieties rich in carbon, nitrogen, and oxygen. The goal of this work is to introduce the same functionalities in isomeric form and understand how the distribution of electron clouds influences the photophysical properties of the resultant CDs. Herein, we investigated the role of the N position in the amine ring of the precursor for CD preparation and its effect on the fluorescence properties of the synthesized nanoparticles (NPs). CDs from diamino pyridine were derived in a facile one pot hydrothermal synthesis. Fluorescence measurements revealed the typical excitation-dependent emission of these NPs but with various multiplicities of the emission centers and different Stokes shifts.

They were subsequently investigated for their size distribution by TEM and AFM, and it was concluded that the photophysical properties were independent of the size of the NPs. Multiple chemical characterization techniques revealed that the chemical compositions of these NPs were thoroughly different and were dominated by the amine position of diaminopyridine (DAP) and the aromatic heterocycles being formed in the system. Density functional theoretical (DFT) calculations led us to conclude that the para- and meta-positions of the amines in the original precursor isomers resulted in NPs with similar properties based on their molecular electrical potential surface’s properties.^31^ We also concluded that the bulk properties were an ensemble average of the fluorescent properties on the single-particle level and can be effectively engineered by the precise choice of the amine containing precursors.

CDs are emerging candidates for fluorescence-based bioimaging due to their unique properties, e.g., good PL, easy synthesis routes, economical synthesis, inexpensive starting materials, water solubility, small sizes, prominent biocompatibility, and excellent photostability.^32^^–^^34^ As an alternative to quantum dots and organic dyes, CDs are a class of fluorescent carbon nanomaterials used in bioimaging, sensing, and optoelectronics. As a result of their unique optical properties, such as tunable emission, facile synthesis, and low toxicity, CDs have applications in biology, medicine, and the environment.^35^^–^^39^ These properties make them translatable and advantageous over other sensing methods for the detection of oral microbiota under biological environment. The various fluorescent behaviors of these CDs have then been employed for selective discrimination of oral microbiota via a machine learning (ML)-driven analysis. The oral microbiota harbors hundreds of species, and it is the cause of several infectious diseases such as dental caries, periodontitis, tonsillitis, and systemic diseases, e.g., infective endocarditis and diabetes.^40^^–^^42^ The current gold standard method for the oral microbiota discrimination is the identification of bacterial genus and species by 16S rRNA sequencing.^43^^,^^44^ This process involves the bacterial DNA extraction and sequencing, which is a laborious, time-consuming, and expensive strategy. Here for the first time, we propose an array that works on the premise of various emergent functional groups in the CDs from the different isomers responding differently to the surface electronic properties of bacteria.

An efficient pattern recognition tool known as linear discriminant analysis (LDA) has been utilized for selective oral bacteria identification based on the fluorescence response of the built array.^45^^,^^46^ This method is a supervised statistical method for classification and intends to maximize the difference between within-class and between-class variations.^47^ The developed array was able to rapidly and accurately identify the type of bacteria and was substantiated from DFT calculations and docking results. The current technology can even identify different types of bacteria in a blended sample of oral microbiota, which could aid dentists in making informed decisions about the potential treatment for the dental diseases.

## 2 Materials and Methods

### 2.1 Materials

Urea, 2,5-DAP, and 2,6-DAP were purchased from Sigma Aldrich (St. Louis, Missouri, United States). 2,4-DAP was from AbaChemScene, LLC (Monmouth Junction, New Jersey, United States). 2,3-DAP and 3,4-DAP were procured from 1 ClickChemistry Inc. (Kendall Park, New Jersey, United States). Nanopure water ($0.2\times 10^{-6}\;m$, 18 MΩ cm) was used throughout the experiments unless noted otherwise. Ward’s^®^ live *Staphylococcus epidermidis* culture, Ward’s^®^ live *Streptococcus mutans* culture, Ward’s^®^ live *Lactobacillus casei* culture, Ward’s^®^ live *Streptococcus salivarius* culture, and *Streptococcus sanguinis* were bought from VWR (Chicago, Illinois, United States) and were subcultured in BHI broth or BHI agar, which were both obtained from Becton Dickinson (Franklin Lakes, New Jersey, United States).

### 2.2 CD Synthesis and Characterization

The CDs from were obtained by dissolving 100 mg DAP and 180 mg urea in 28 ml water. The mixture was then transferred to an autoclave synthesizer, and it was heated for 2 h at 180°C. Care was taken to carry out synthesis in a single autoclave chamber to minimize the effect of cooling rate on various NPs. The as-obtained suspensions were sonicated for X min using a tip sonicator (Q700, Qsonica Sonicators, Newtown, Connecticut, United States) operating at a pulsed amplitude of 1 with a cycle of 2 s on and 1 s off. Finally, the samples were passed through syringe filters with filter sizes of 0.45 and 0.22  μm (Biomed Scientific) before being lyophilizing. The lyophilized powders were stored in the fridge (4°C) and were dispersed in water before each experiment after mild sonication.

For the transmission electron microscopy analysis, the NPs were deposited on QUANTIFOIL^®^ holey carbon films (Electron Microscopy Sciences, Hatfield, Pennsylvania, United States), and then water was wicked away by filter paper. The samples were inspected by TEM (FEI Company, Hillsboro, Oregon, United States) using an instrument equipped with a Peltiercooled Tietz (TVIPS) 2k×2k charge-coupled device camera with an operating voltage of 120 kV. The anhydrous diameter was determined using image J software (NIH, Bethesda, Maryland, United States).

For AFM imaging, the NPs were drop cast on mica mounted on a steel disk, and the images were acquired on a Bruker MultiMode Nanoscope IIIA (Billerica, Massachusetts, United States). The images were analyzed by Gwyddion, and the height profile was constructed using the “extract profile” option after leveling the data and removing scars.

The electrophoretic ζ potentials were measured by the Malvern Zeta Sizer ZS90 instrument (Malvern Instruments Ltd., United Kingdom).

Raman spectra of DAP NPs were collected on a Nanophoton RAMAN 11 laser Raman microscope (Osaka, Japan) equipped with an excitation laser of 532 nm. The grating was set to $600\;g\cdot {\mathrm{mm}}^{-1}$.

Fourier transform infrared spectroscopy (FTIR) was carried out on the samples dried on MirrIR IR-reflective glass slides (Kevley Technologies, Chesterland, Ohio, United Staes). Nicolet Nexus 670 FT-IR (MRL Facility, UIUC) was utilized for measurement in the attenuated total reflectance (ATR) mode.

X-ray photoelectron spectroscopy (XPS) was performed on a Physical Electronics PHI 5400 spectrometer using Al Kα (1486.6 eV) radiation. The samples were dried in a vacuum oven for 24 h to remove residual water. The analysis of the peaks and calibration with adventitious C─C bond (284.8 eV) were done with CasaXPS software. The mass spectra in ESI mode were completed at the mass spec facilities at UIUC.

The photophysical characteristics of the NPs were characterized by several methods. The absorbance spectra were collected using a GENESYS 10 UV–Vis spectrometer (Thermo Scientific, Massachusetts, United States) on a sample dispersed in phosphate buffer saline (pH = 7.4). The emission spectra were recorded on an Infinite 200 PRO multimode microplate reader (Tecan, North Carolina, United States) at an excitation wavelength of 360 nm in the dark well plates (Falcon black/clear sterile 96-well imaging plate). The 2D excitation emission contours were measured on Horiba Aqualog Scanning Spectrofluorometer (Horiba scientific, Edison, New Jersey). The first-order Rayleigh scattering was corrected, and all spectra were normalized to $1\;{\mathrm{mg}\cdot L}^{-1}$ quinine sulfate.

PL lifetime data were acquired using a home-built setup based on a NKT SuperK and a doubled Spectra Physics Mai Tai lasers, a SP500 Princeton Instruments spectrometer with a Pixis CCD camera, an ID Quantique ID100 single-photon avalanche detector, and a Becker and Hickl SPC-130 time-correlated single-photon counting module giving a time resolution <100  ps. The laser excitations at 390 and 510 nm were utilized.

Origin software was used to obtain the decay time and the average decay time for NPs with multiexponential decay. The photoluminescence decay curves were fitted with the following function:

$$I(t)=A_{1}e^{-t/\tau_{1}}+A_{2}e^{-t/\tau_{2}}\;\text{and}\;B_{1}+B_{2}=1.$$

The amplitude weighted average lifetime of the entire photoluminescence was calculated as follows:

$$\tau_{\text{average}}=\frac{\sum_{i=1}^{i=n}\alpha_{i}\tau_{i}^{2}}{\sum_{i=1}^{n=1}\alpha_{i}\tau_{i}},$$

where $\alpha_{i}$ denotes the preexponential factor and $\tau_{i}$ is the i’th decay time.

The relative quantum yield (QY) was calculated with respect to quinine sulfate in 0.1 N $H_{2}{\mathrm{SO}}_{4}$, which has a known QY of 53%. The CDs concentration was adjusted to obtain ${\mathrm{OD}}_{365}$ (i.e., absorbance at 365 nm with pathlength = 1 cm) <0.1. Subsequently, six dilutions were made, each having half of the concentration of the previous step. A similar procedure was followed for quinine sulfate. These samples were further subjected to fluorescence spectroscopy analysis with $\lambda_{\mathrm{ex}}=365\;\mathrm{nm}$, and their emission fluorescence intensity was integrated using Origin software. The integrated fluorescent intensity versus absorbance (m) curves were constructed, and the slopes of the curves were exploited to obtain the QY as follows:

$$\varphi_{\mathrm{CD}}=\varphi_{\mathrm{QS}}.\frac{m_{\mathrm{CD}}}{m_{\mathrm{QS}}}\times \frac{\mu_{\mathrm{CD}}}{\mu_{\mathrm{QS}}}.$$

The refractive indices (μ) for both the CDs and quinine sulfate solution were considered to be 1.33.

### 2.3 Theoretical Calculations

The molecules were drawn in Chem3D CambridgeSoft and were saved in .gjf for further processing in Gaussian09 software package. The time-dependent density functional theory was implemented on the 6-311G(d) Pople basis set combined with Becke three-parameter hybrid density (B3LYP) functional in vacuum to obtain the optical properties. The bandgap energy was calculated based on the difference in the energy of the highest occupied molecular orbital (HOMO) and lowest unoccupied molecular orbital (LUMO). The surface rendering of frontier molecular orbitals was done on VMD software. The blue and red colors in the molecular orbitals demonstrate the negative and positive phases of the HOMO and LUMO wavefunctions.

### 2.4 Single-Particle Imaging

Objective-based total internal reflection fluorescence microscopy (TIRFM) was used for single-particle imaging. A continuous wavelength (488 nm, Spectral physics) laser was used as the light source. An inverted microscope (IX73) equipped with a 100× oil immersion objective (Olympus, PlanApo, 100×, NA 1.49, oil immersion) was used. The laser beam was then expanded, collimated to about 35 mm, and directed into the microscope by 400 mm lens (Thorlabs LA1725A). The incident light was directed through the objective via an exciter (FF01-482/563-25, Semrock) and a dual-band dichroic filter (Di01-R488/561-25 × 36, Semrock). The mean excitation power before the objective is about $1.5\;\mathrm{mW}\;{\mathrm{cm}}^{-2}$. The luminescence photons from individual CDs were collected by the same objective, passed an emitter (FF01-523/610-25, Semrock), and captured by an electron multiplying charge coupled device (EMCCD) camera (iXon U797, Andor. Technology). The emission filter was designed to have a spectral cutoff at 500 to 548 nm, covering the central wavelength of the CDs emission at 500 nm. Thus experimental observations of emission intensity were not affected by potential spectral diffusion of ±10  nm. The sample coverslip was secured onto a 2D stage. Individual NPs were located by raster scanning the stage. An exposure time of 100 ms was used. At least ten time-stamped image stacks, each of which consisted of 600 frames, were taken for each type of particle.

### 2.5 Image Analysis for Single-Particle Imaging

The NPs were deposited on a cover slip and were dried prior to imaging. We followed the protocols from our laboratories published previously.^48^^–^^50^ A homemade objective-based TIRFM equipped with an EMCCD camera was utilized for this experiment (100× oil immersion objective, n=1.49, power = 1.5 mW, $\lambda_{\mathrm{ex}}=488\;\mathrm{nm}$). For the bleaching experiment, the field of view (FOV) was adjusted to 80  μm×80  μm with a total acquisition time of 8 min per FOV with 200 ms exposure time per frame. The analysis was done in a custom-written MATLAB code that accounts for the background as the average intensity of peripheral pixel in a 7×7  pixel area minus the central spot. The bleaching curve was constructed by plotting the normalized number of particles versus time and subsequently fitting the results to a two-component exponential function.

### 2.6 Bacterial Separation Sensor Array

Several strains of dental biofilm causing bacteria were grown overnight for 24 h, and their concentrations were adjusted to $10^{6}\;\mathrm{CFU}\;{\mathrm{ml}}^{-1}$ and plated in 96 well plates. Subsequently, they were incubated with $1\;\mathrm{mg}\;{\mathrm{ml}}^{-1}$ of NPs (diluted from $100\;\mathrm{mg}\;{\mathrm{ml}}^{-1}$ stock), and the bacteria was grown in the microplate reader with a temperature that was adjusted to 37°C. The fluorescence was measured at $\lambda_{\mathrm{ex}}=360\pm 40\;\mathrm{nm}$ and $\lambda_{\mathrm{em}}=460\pm 40\;\mathrm{nm}$ every 5 min up to 30 min. Water was chosen as a positive control. The difference in the fluorescence intensity and the growth time constant were fed to the ML algorithms to generate the bacterial types using MATLAB.

### 2.7 Docking Studies

The CDs were modeled as different ovalene derivatives,^48^ and the chemical structures were energy minimized using a general *ab initio* quantum chemistry package, the general atomic and molecular electronic structure system (GAMESS) program.^51^ We used B3LYP functional while performing the DFT calculations with 6-31G(d) as the basis set.^52^ Pople N31 was used for the polar groups. These energy minimized structures were then undertaken for docking studies^53^ using AutoDock 4.0 software.^54^

### 2.8 HOMO–LUMO Calculations

The chemical structures were initially energy optimized, and the HOMO–LUMO surfaces were then calculated from their energy minimized geometries using GAMESS. We used B3LYP functional while performing the DFT calculations with 6-31G(d) as the basis set. Pople N31 was used for the polar groups. The HOMO and the LUMO were calculated from the energy minimized geometries.

## 3 Results and Discussion

We created a library of CDs based on DAP via the cocarbonization of DAP compounds with urea in a hydrothermal method. In a typical synthesis, 0.18 g of each DAP isomer was mixed with 0.1 g urea and dissolved in 28 ml of water. The mixture was then transferred to an autoclave vessel for nucleation at 180°C for 2 h. Care was taken to minimize the systematic errors using the same autoclave vessel for all five samples and with the same cooling rate. The NPs were then filtered, dialyzed against water (sink condition), and freeze dried for subsequent use.

**Fig. 1:**
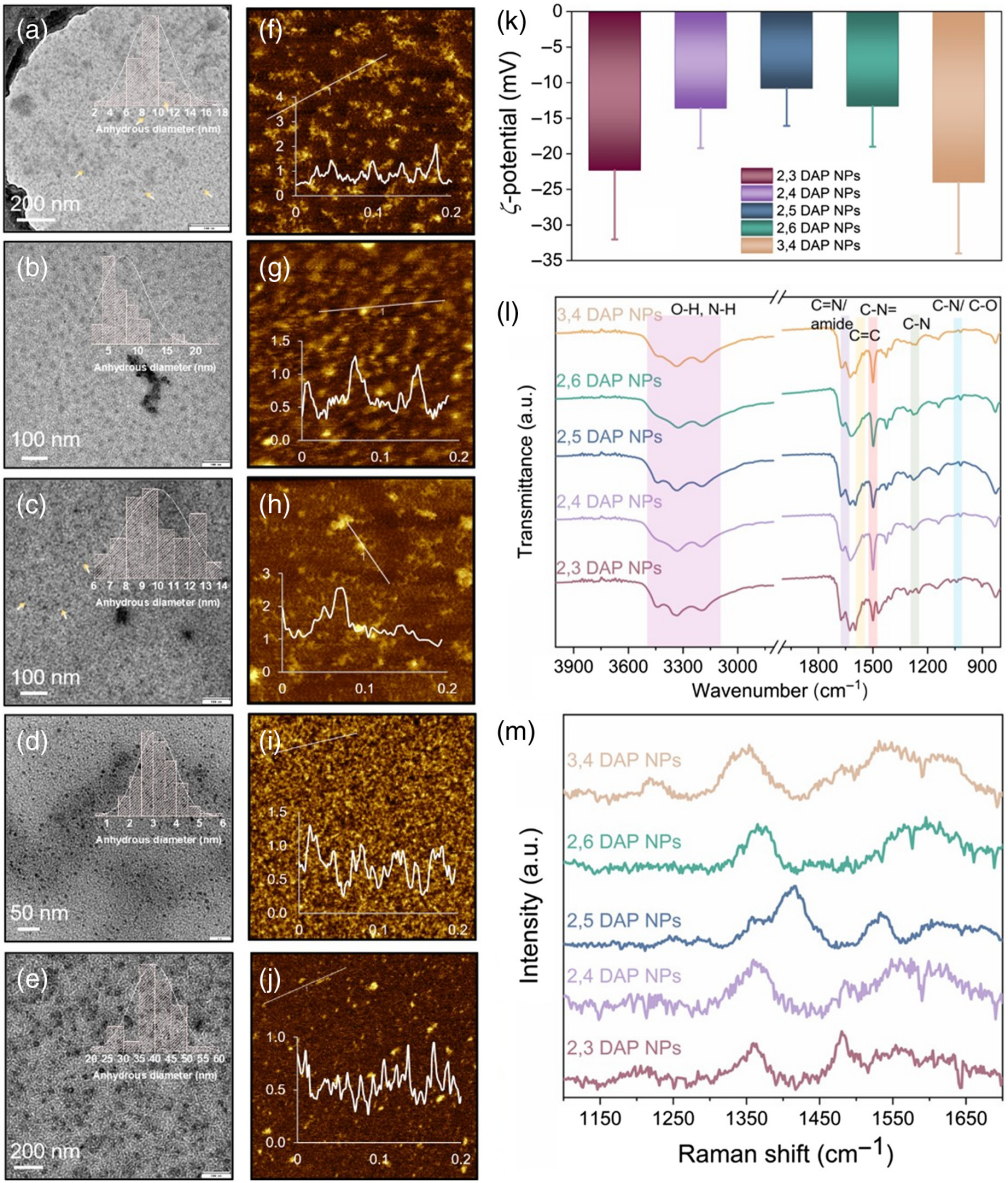
Physiochemical characterizations of DAP NPs: TEM images of (a) 2,3 DAP NPs, (b) 2,4 DAP NPs, (c) 2,5 DAP NPs, (d) 2,6 DAP NPs, (e) 3,4 DAP NPs; AFM images and the height distribution for (f) 2,3 DAP NPs, (g) 2,4 DAP NPs, (h) 2,5 DAP NPs, (i) 2,6 DAP NPs, (j) 3,4 DAP NPs; (k) electrophoretic zeta potential; (l) FTIR; and (m) Raman spectra of NPs.

The transmission electron microscopy (TEM) and atomic force microscopy (AFM) of these NPs were carried out as shown in Figs. 1(a)–1(e). The transmission electron microscopy indicated the presence of spherical particles. It was realized that the anhydrous size of 2,3 DAP NPs, 2,4 DAP NPs, 2,5 DAP NPs, and 2,6 DAP NPs are almost similar and measured as 9±3, 8±4, 10±2, and 3±1  nm, respectively, whereas for 3,4 DAP NPs, the anhydrous diameter was much bigger and measured as 40±7  nm [Figs. 1(f)–1(j)]. These CDs of <10  nm in diameter generally have lower contrast than other high density, high atomic number metallic NPs under TEM owing to the weak electron scattering cross section of carbon. Additionally, none of these particles contain heavy atoms or are stained with heavy elements. As a result, TEM images of them tend to be low contrast. On the other hand, the AFM height measurement yielded similar results for all NPs, possibly due to the flattening of the NPs on the surface of mica.

Subsequently, the electrophoretic ζ-potential showed negative values for all prepared CDs [Fig. 1(k)]. The absolute high zeta potential values (∼10  mV) pertain to the reasonable colloidal stability of these NPs, which originate from the replete hydrophilic surface functional groups as was concluded from FTIR.

FTIR, as a powerful tool in determining the surface functional groups, was utilized [Fig. 1(l)]. The CD surface presented an abundance of polar functional groups with a strong band observed between 3100 and $3500\;{\mathrm{cm}}^{-1}$ due to the stretching vibration of the polar functional group in O─H and N─H, with the peak centered at $1131\;{\mathrm{cm}}^{-1}$ due to C─O. On the other hand, C═C, which is typical of aromatic structures appeared, between 1539 and $1635\;{\mathrm{cm}}^{-1}$. The peak centered at $1673\;{\mathrm{cm}}^{-1}$ could be related to amide and/ or C═N as result of graphitic N, whereas the strong peak centered at $1497\;{\mathrm{cm}}^{-1}$ could be ascribed to C─N═. Finally, the asymmetric stretching vibration of C─N and C─O could be detected at 1127 to $1159\;{\mathrm{cm}}^{-1}$.

Subsequently, Raman spectroscopy was utilized to distinguish the D and G bands, which typically appear for CDs [Fig. 1(m)]. The D-band (diamond-band) ($1355\;{\mathrm{cm}}^{-1}$), or the disorder band, occurs as a result of the out of plane vibration of ${\mathrm{sp}}^{2}$ C in a pool of ${\mathrm{sp}}^{3}$ molecular defect states. On the other hand, the G band ($1605\;{\mathrm{cm}}^{-1}$), or the graphitic band, corresponds to E2g mode from the in-plane vibration in ${\mathrm{sp}}^{2}$ C within the aromatic domain. The ratio of D/G band was calculated, and it turned out that the D/G ratio is 2,6 DAP NPs (0.92) < 3,4 DAP NPs (1.10) < 2,5 DAP NPs (1.14) < 2,4 DAP NPs (1.18) < 2,3 DAP NPs (1.37). The increase in the D/G ratio implies the lowering of the crystallinity, meaning that 2,6 DAP NPS possesses the largest ${\mathrm{sp}}^{2}$ domain.

**Fig. 2:**
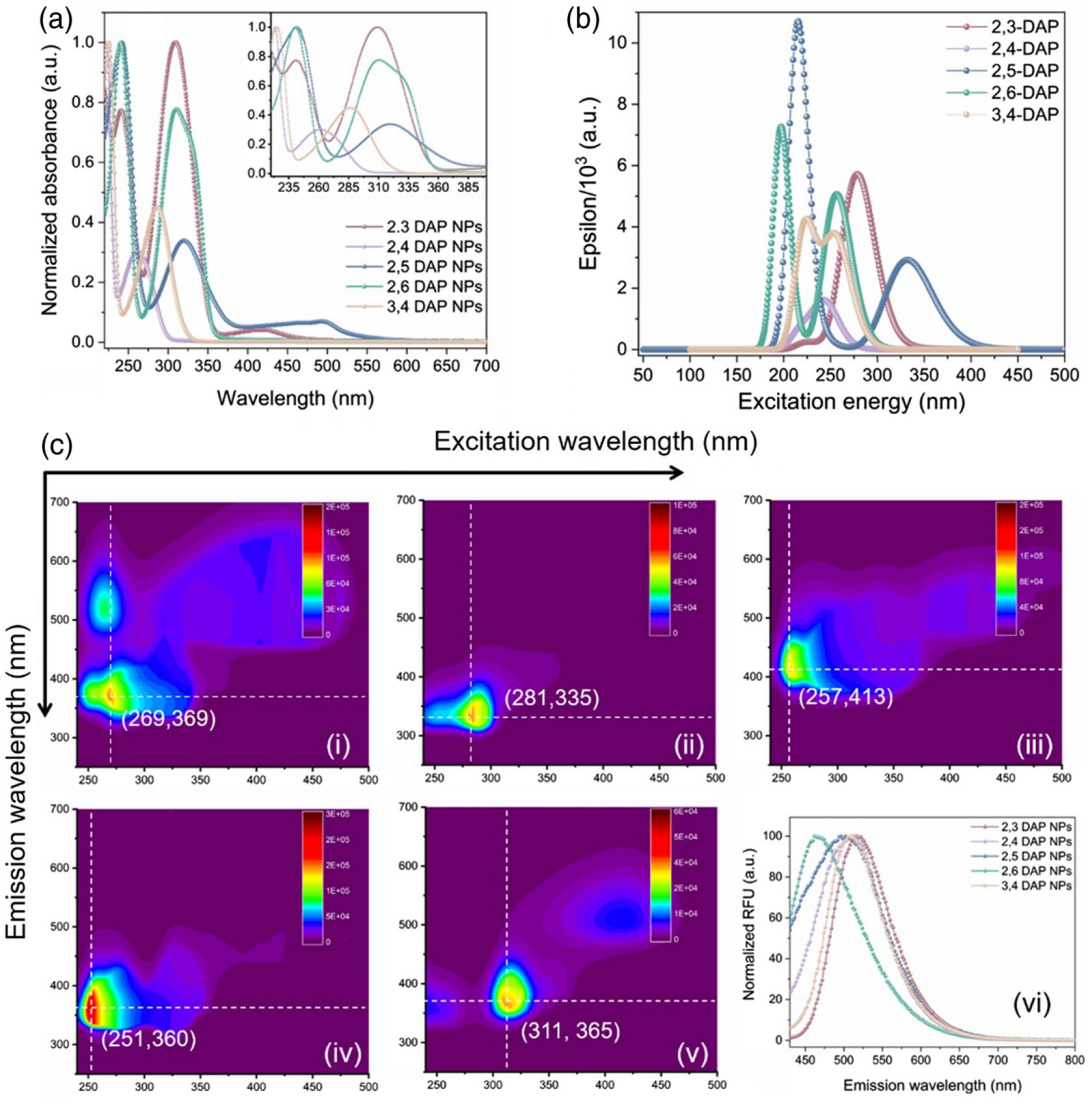
(a) Experimental UV–Vis spectra of various NPs and the inset indicates the magnified region between 233 and 390 nm. (b) The predicted spectra of DAP molecules based on DFT calculations. (c) 2D fluorescence map for (i) 2,3 DAP NPs, (ii) 2,4 DAP NPs, (iii) 2,5 DAP NPs, (iv) 2,6 DAP NPs, (v) 3,4 DAP NPs, the emissions centers are identified; and (vi) the normalized emission fluorescent spectra of NPs excited at 360 nm.

We performed UV–Vis spectroscopy to determine the absorbance associated with these NPs [Fig. 2(a)]. For all NPs, at least two absorbance states could be identified: the core state occurring in the UV region and a surface state. The core state, which is attributed to π→π* transition in the aromatic hydrocarbon domains, of 2,4 DAP NPs, and 3,4 DAP NPs appeared in the high-energy UV band, whereas 2,3 DAP NPs, 2,6 DAP NPs, and 2,5 DAP NPs underwent a bathochromic shift. On the other hand, the surface state, which indicates a transition between n-orbital of heteroatom (i.e., oxygen and nitrogen) and the π* of polyaromatic core, appeared in the following order of wavelengths: 2,4 DAP NPs< 3,4 DAP NPs < 2,3 DAP NPs = 2,6 DAP NPs < 2,5 DAP NPs. Interestingly, a second surface state is attributed to a lower extinction coefficient for 2,3 DAP NPs and 2,5 DAP NPs, which is red shifted in 2,5 DAP NPs. This exitonic band could be due to lower energy species on the surface of CDs, which resulted in narrowing of the electronic band gap in these two cases. These results match well with the simulated UV–Vis results simulated from DFT calculations of the original precursors as indicated in Fig. 2(b). For better understanding, the experimental and theoretical (from DFT) UV–Vis spectra are plotted separately in Fig. S1 in the Supplementary Material.

Figure 2(c) demonstrates the 2D excitation–emission photoluminescence maps for various CDs obtained from DAP. From an asymmetric shape of excitation–emission centers, an excitation-dependent emission phenomenon usually reported in CDs that disobeys the Kasha–Vavilov’s rule can be concluded.^55^ The red edge excitation shift might be due to the radiative recombination by surface traps caused by various surface functional groups. The emission centers for the NPs were determined as follows: for 3,4 DAP NPs (311 and 365 nm) with the Stokes’ shift value of $4735.45\;{\mathrm{cm}}^{-1}$, for 2,5 DAP NPs (257 and 413 nm) with the Stoke’s shift value of $14,691\;{\mathrm{cm}}^{-1}$, for 2,4 DAP NPs (281 and 335 nm) with the Stoke’s shift of $5742\;{\mathrm{cm}}^{-1}$, for 2,6 DAP NPs (251 and 431 nm) with the Stoke’s shift of $16,656\;{\mathrm{cm}}^{-1}$, and for 2,3 DAP NPs at (251 and 360 nm) with the Stoke’s shift of $12,063\;{\mathrm{cm}}^{-1}$. The difference between emission maxima and excitation maxima followed this trend: 2,6 DAP NPs < 2,5 DAP NPs < 2,3 DAP-NPs < 2,4 DAP NPs < 3,4 DAP NPs. The observed behavior can be justified based on the chemical properties of these NPs as was elucidated by XPS and mass spectroscopy (mass spec), which suggest that the photoluminescence is governed by a combination of both core states and the fluorophores at the edge state.

**Fig. 3:**
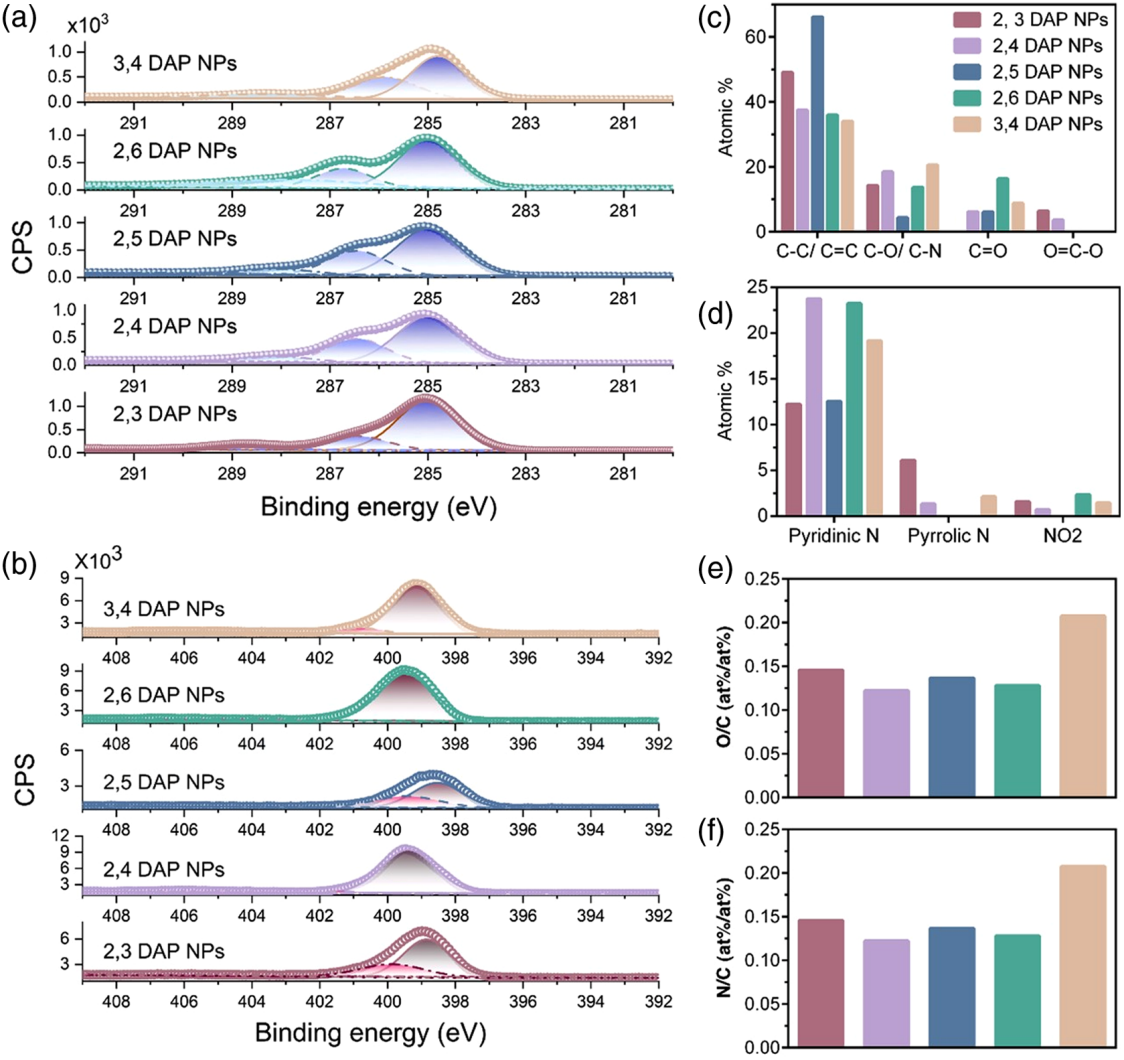
Deconvoluted (a) C1s region and (b) N1s region; (c) the comparison of various C-containing functional groups; (d) N-containing functional groups; and (e) the O/C and (f) N/C atomic percent distribution for different NPs.

XPS aided us in identifying surface functional bonds, and the presence of C, N, and O was detected in the survey spectra with peaks at 285, 400, and 531 eV, respectively, (Figs. S2–S11 in the Supplementary Material). The carbon adventitious peak was corrected to 284.8 eV, and the C1 and N1S are shown in Figs. 3(a) and 3(b), respectively. The atomic percentage ratio of O/C and N/C is shown in Figs. 3(e) and 3(f), and it was determined that 3,4 DAP NPs has the highest level of oxidized species while also possessing the highest N/C doping. The decomposition of C1s core state revealed the presence of C─C/C═C at 284.8 eV, C─O/C─N at 286.3 eV, carbonyl group ─C═O at 288.1, and ─COOH at 289.9 eV [Fig. 3(c)]. The binding energies in N1s region [Fig. 3(d)] at 398.9, 400.5, 401, and 404.5 eV were attributed to pyridinic nitrogen, pyrrolic nitrogen, and nitro, respectively. The O1s decomposition also revealed the presence of C─O and C═O at 534.4 and 532.1 eV, respectively. 2,5 DAP NPs has the most ${\mathrm{sp}}^{2}$ C followed by 2,3 DAP NPs, whereas the rest seem to have almost the same amount. 2,5 DAP NPs has the least ${\mathrm{sp}}^{3}$ C followed by 2,6 DAP NPs = 2,3 DAP NPs and then 3,4 DAP NPs = 2,4 DAP NPs, whereas 2,6 DAP NPs has the highest carbonyl amount; 2,3 DAP NPs and 2,4 DAP NPs are the only species having the carboxyl group.

The N in the CDs can exist in various forms, and it can drastically affect the fluorescent behavior. In these cases, N substitutes for the C atom in the polyaromatic cyclic structure, leading to the σ-bond formation. On the other hand, the aromatic π bond forms and the fifth electron enters the antibonding π* molecular orbital to form either a six- or five-member ring. This leads to the creation of pyridinic and pyrrolic moieties that affect the electronic properties of CDs. Pyrrolic N enhances fluorescence, whereas pyridinic N inhibits it due to the greater distortion that is caused by the five-membered ring. Pyridinic N causes more bathochromic-shift than pyrrolic N due to the contribution of the lone pair electron in the π-system aka delocalized electron. This freely moving electron has various wavefunctions, which can result in the narrowing of the π→π* transition and hence a red-shift in the spectra.^56^ Interestingly, graphitic nitrogen, which is known to trigger the red shift, is absent in the XPS spectra, which could suggest that the fluorescent is originating from the doped fluorophores formed during the synthesis rather than the core state. More importantly, the amine N, which also highly contribute to the red-shift in the CDs, are missing as determined by XPS, which could be due to full oxidation of the surface exposed amines.^57^ Because XPS is a surface characterization technique, to get more insight into the composition of these NPs and understand their photophysical properties, which is not immediately apparent by other methods, we sought structural elucidation by mass spectroscopy analysis.

**Fig. 4:**
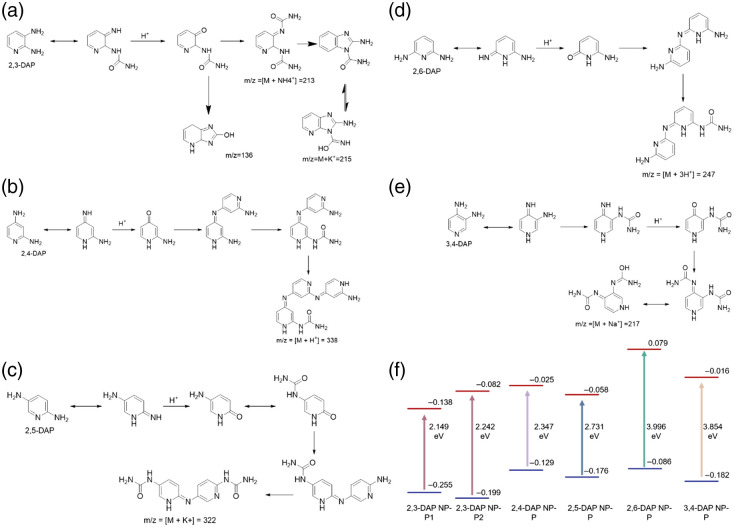
(a)–(e) The predicted molecules based on mass-spec results and (f) the band gap energy and the HOMO–LUMO energy. Note that molecular orbital energies are in Hartree Fock (HF).

The predicted molecules corresponding to the m/z values detected in mass spectroscopy and the proposed mechanism for the production of these molecules are shown in Figs. 4(a)–4(e). Furthermore, we did further time-dependent DFT calculations to numerically determine the bandgap values and the distribution of the frontier orbitals in the predicted products, as is shown in Fig. 4(f). From these mechanisms combined with theoretical calculations, several points can be concluded and can be correlated with the observed PL behavior.

First, the steric effect occurs between two amino groups in both the 2,3 DAP and 3,4 DAP compounds due to the presence of two amino groups in the ortho position. Both compounds showed similar fluorescence properties due to the similarities of their structural arrangements. However, more red shifted emission occurs for 2,3 DAP NPs than 3,4 DAP NPs, which can be explained by the presence of the imidazole ring that is formed during the reaction between diamino pyridine and urea in the thermal condition. The bandgap value was calculated to be 2.15 and 2.24 eV for molecules 2,3 DAP NP-P1 and 2,3 DAP NP-P2, respectively, which is the smallest bandgap among all of the predicted molecules. Second, the more blue shifted emission observed for 2,6 DAP NPs among other CDs can be explained due to the presence of highest band gap energy between HOMO and LUMO (3.99 eV). On the other hand, the 2,5 DAP NPs product with a bandgap of 2.73 eV showed a red shifted emission due to extended conjugation occurring as both amine groups are in para-position. Next, 2,4 DAP NPs showed a slightly more red shifted emission than 2,5 DAP NPs. This fluorescence behavior was due to two reasons: (i) the less band gap energy between HOMO and LUMO (2.35 eV) and (ii) the presence of more conjugation because of the involvement of a greater number of DAP molecules to get the final products.

**Fig. 5:**
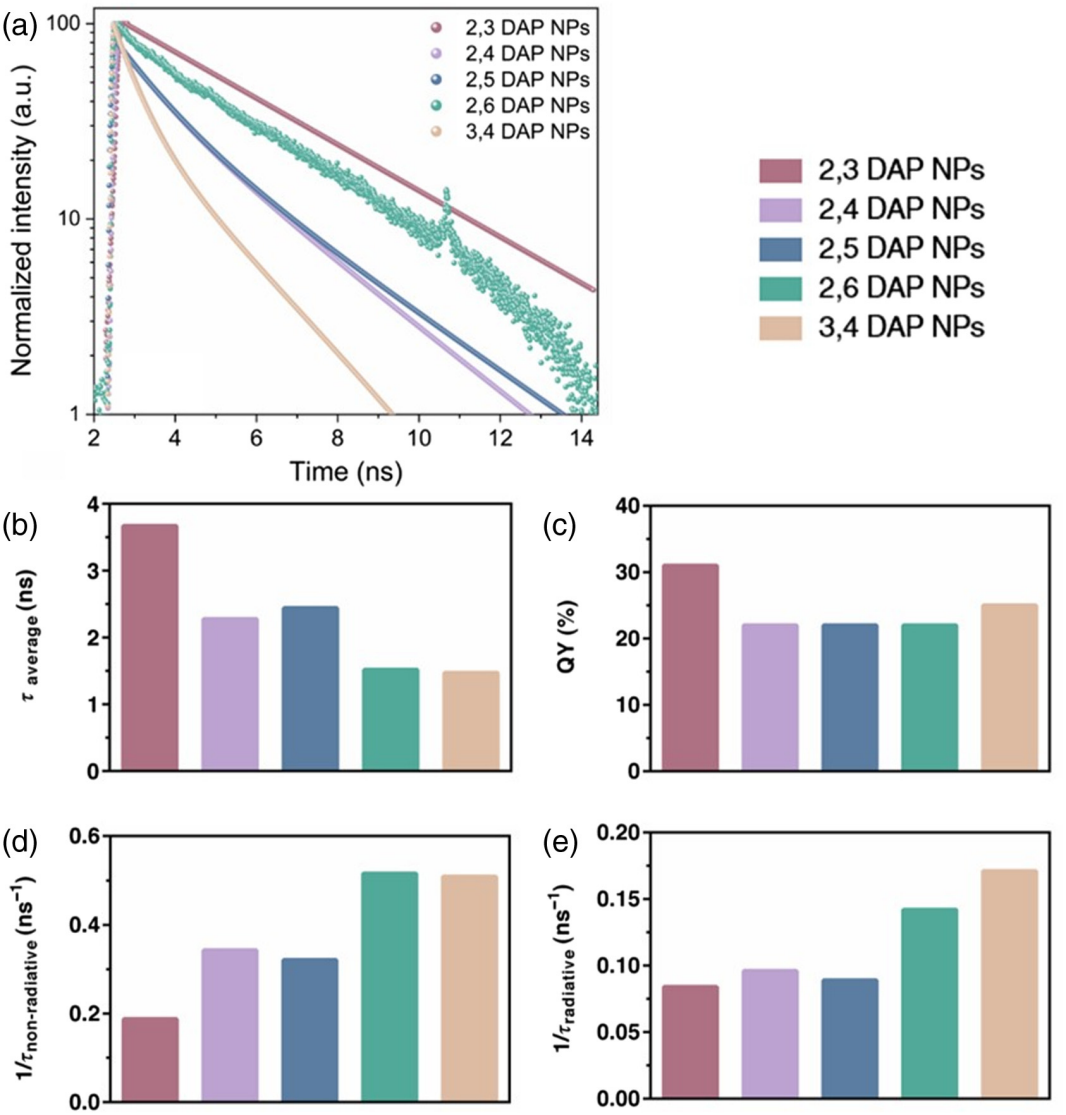
(a) TRPL spectra of NPs with $\lambda_{\mathrm{exc}}=390\;\mathrm{nm}$; (b) amplitude weighted average decay lifetime; (c) %quantum yield (QY) values for the five different DAP nanoparticles synthesized herein; (d) non-radiative decay rate; and (e) radiative decay rate.

Time-resolved photoluminescence (TRPL) spectroscopy was conducted with lasers at 390 nm [Fig. 5(a)] and 510 nm (Fig. S12 in the Supplementary Material), which correspond to the excitation of both the core and surface states, respectively. The NPs indicated double exponential decay when excited at both 390 and 510 nm, suggesting the presence of multiple emission centers on the surface and hence multiple radiative recombination pathways involved in this phenomenon. The photoluminescence of CD is still a matter of debate, and it has been attributed to various mechanisms, including the quantum size effects, surface traps, and intrinsic state.^58^^–^^60^ CDs are comprised of a carbongenic ${\mathrm{sp}}^{2}$ core, whereas the surface is replete with surface functional groups that mediate the fluorescence properties.^61^ Herein, the relaxation of the photoexcited electrons during π→π* transition can result in two exponential decay components: the first decay, i.e., $\tau_{1}$, arises from the molecular state ${\mathrm{sp}}^{2}$ relaxation, whereas $\tau_{2}$ originates from the heterogeneous surface state.^48^ The amplitude weighted average decay lifetime $\tau_{\text{average}}$ is shown in Fig. 5(b). The difference in $\tau_{\text{average}}$ can be attributed to the density, nature, and orientation of the surface functional groups, which directly affect the charge carrier trapping and excitation relaxation pathways as has been concluded from the XPS results. On the other hand, the information about mass spectroscopy led us to similar conclusions. In 2,3 DAP NPs, the radiative decay mechanisms were more well-defined, leading to a slower decay time. It is also interesting to note that the average lifetime was shorter in TRPL when excited at 510 nm compared with 390 nm, suggesting that the surface state has a more fleeting relaxation time compared with the core state.

Furthermore, the PL QY was obtained by comparing the absorbance (365 nm) over fluorescence integral of the NPs ($\lambda_{\mathrm{ex}}=365\;\mathrm{nm}$) versus the similar parameter for quinine sulfate dissolved in 0.1 N $H_{2}{\mathrm{SO}}_{4}$ (OD<0.1) with a known QY = 0.53 [Fig. 5(c)]. The calculated relative QY for 2,3 DAP NPS, 3,4 DAP NPs, 2,4 DAP NPs, 2,5 DAP NPs, and 2,6 DAP NPs was 31%, 25%, 22%, 22%, and 22%, respectively.

To understand the transfer phenomena governing the photoluminescence and investigate the emission kinetics, we correlated the QY and TRPL results to calculate the radiative and non-radiative decay rate components of the emitters via the following equations:^62^

$$\tau_{\mathrm{PL}}^{-1}\;\tau_{\text{average}}=\frac{1}{k_{R}+k_{\mathrm{NR}}},\;\mathrm{QY}=\tau_{\text{average}}\times k_{R}.$$

By solving the above equations, one can obtain $k_{\mathrm{NR}}$ and $k_{R}$, which stand for non-radiative recombination rate and radiative recombination rate in ${\mathrm{ns}}^{-1}$, respectively [Figs. 5(d) and 5(e)].

**Fig. 6:**
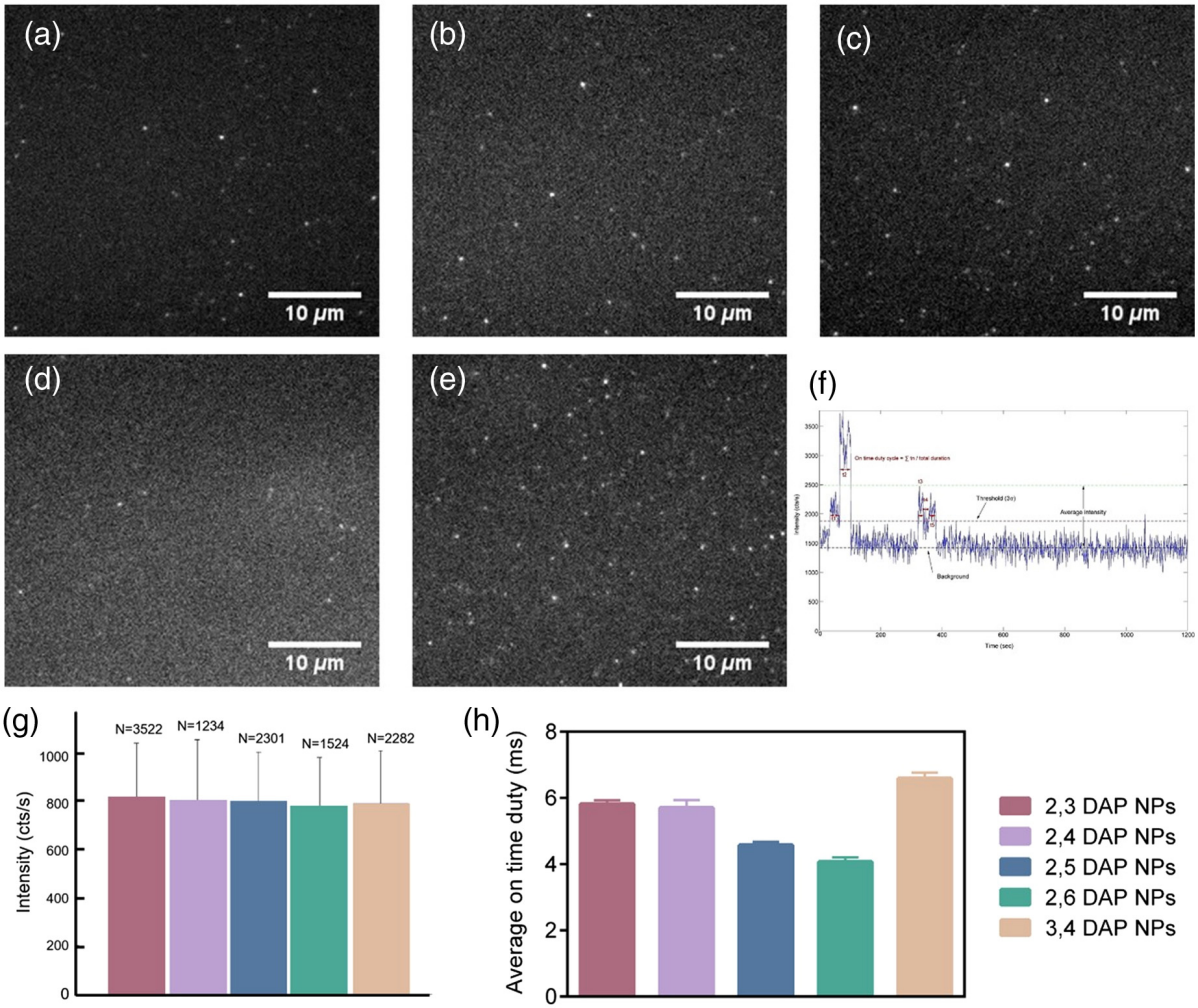
Photophysical properties of DAP NPs on the single-particle level, Representative single-particle emission images of (a) 2,3 DAP NPs, (b) 2,4 DAP NPs, (c) 2,5 DAP NPs, (d) 2,6 DAP NPs, and (e) 3,4 DAP NPs as captured by an EMCCD camera. (f) Representative photoluminescence intensity trajectory indicative of particle brightness, (g) the instantaneous intensity of the NPs, and (h) the average ON duty time (Video 1, MP4, 30.7 MB; [URL: https://doi.org/10.1117/1.JBO.28.8.082807.s1] Video 2, MP4, 30.7 MB [URL: https://doi.org/10.1117/1.JBO.28.8.082807.s2] Video 3, MP4, 30.7 MB; [URL: https://doi.org/10.1117/1.JBO.28.8.082807.s3] Video 4, MP4, 30.7 MB; [URL: https://doi.org/10.1117/1.JBO.28.8.082807.s4] Video 5, MP4, 30.7 MB; [URL: https://doi.org/10.1117/1.JBO.28.8.082807.s5]).

To further elucidate the photophysical properties, we investigated the photo-blinking state of CDs using single-particle imaging in the TIRF microscopy [Figs. 6(a)–6(e)].^48^ Figure 6(f) demonstrates an example of intensity time trace of X NPs, indicating a multistep blinking that could be attributed to multiple emissive centers on the CDs. The blinking of CDs was conceived with the characteristic on and off states as identified by intensity thresholding with the mean±3x standard deviation (σ).^49^^,^^63^ The intermittent and stochastic nature of these CDs with bright spots at certain time points are indicated in time lapse image movies (Videos 1, 2, 3, 4, and 5; see Fig. 6 caption). Two parameters are used to determine the level of the brightness of DAP NPs: instantaneous intensity and duty cycle. The instantaneous intensity was determined to be quite similar for all of the DAP NPs [Fig. 6(g)]. The average of finding a particle in the emissive state during the acquisition cycle of NPs was determined and indicated in Fig. 6(f). On the other hand, the average duty cycle seems to vary between various NPs [Fig. 6(h)]. The origin in the brightness between different species was not clear but could possibly be due to the various trap state distributions on the CDs.

In addition, we carried out bleaching experiments to determine the photostability of the CDs. All NPs presented a single-step bleaching time that can accordingly be attributed to the presence of highly coupled chromophores previously noted in the conjugated polymers (Figs. S18–S22 in the Supplementary Material).^64^^–^^66^

**Fig. 7:**
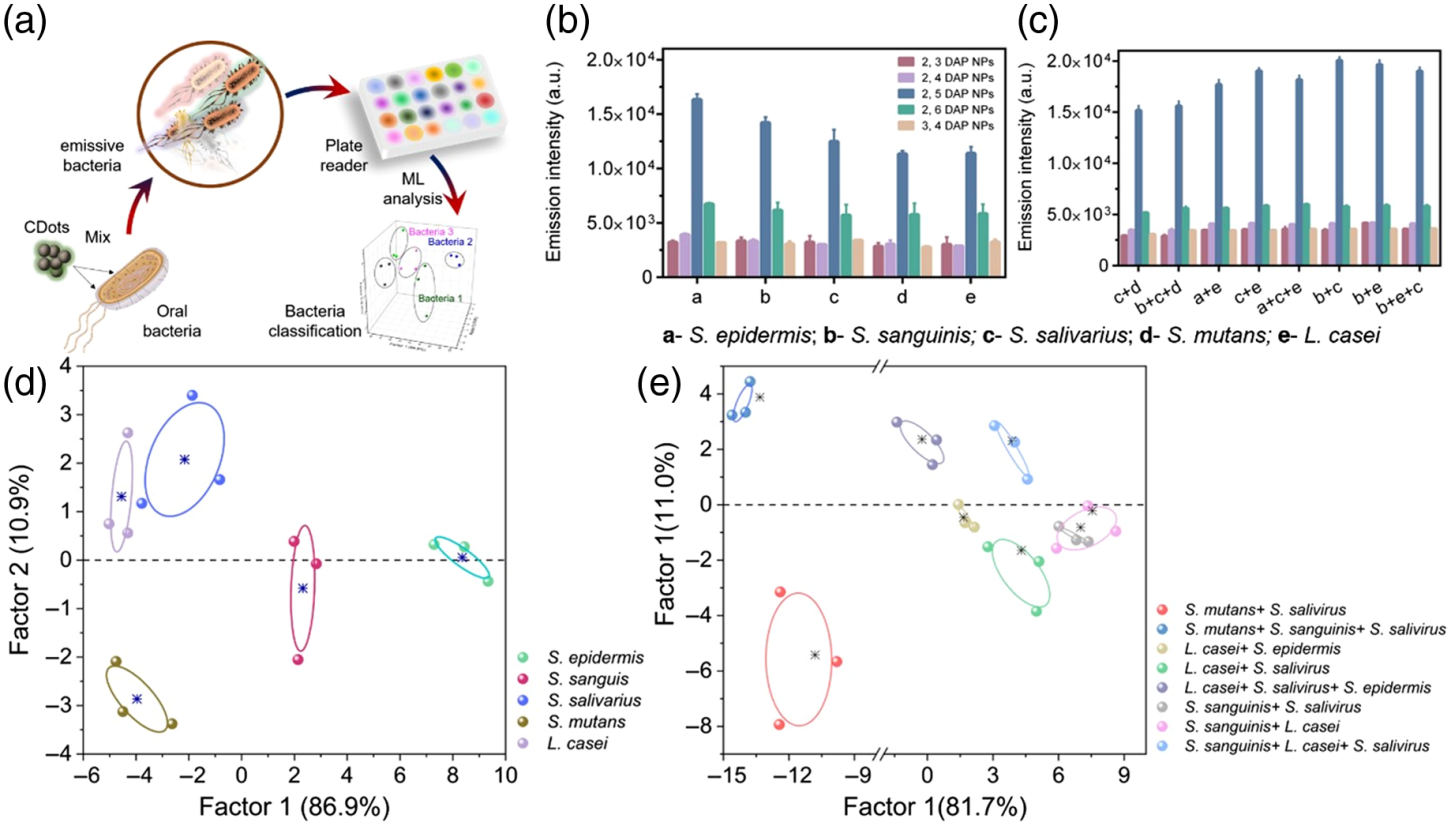
(a) Schematic illustration of sensor array from 5 different NPs and oral bacterial separation based on ML tools. The fluorescence output of NPs when exposed to (b) single species and (c) coculture of bacteria. (d) LDA classification of single bacterial strain and (e) a coculture of several bacterial strains achieving 100% separation.

Thus having unraveled the underpinning PL mechanisms mediated by the precursor’s composition, five DAP NPs were utilized as a building element for the sensor arrays [Fig. 7(a)]. The fluorescence response of the five DAP NPs when interacted with each bacteria represent the fluorescence signature toward each bacterium. The signal from each DAP NPs was found to be unique to the type of bacterium [Fig. 7(b)]. In addition, the synthesized DAP NPs showed a distinguishable signal toward blended samples of bacteria [Fig. 7(c)]. To evaluate the ability of the built sensor array using the five DAP NPs, the fluorescence signatures of each bacteria [Figs. 7(b) and 7(c)] produced by the sensor array were analyzed using the LDA, a powerful statistical method extensively harnessed in pattern recognition.^67^^–^^70^ Using LDA, the fluorescence pattern of the bacteria sample could be transformed to a 2D canonical score [Fig. 7(d)]. First, the model was trained using a training dataset consisting of the fluorescence response to the five oral bacteria. The five oral bacteria were well-clustered into five groups and discriminated completely from each other. Next, we challenged the model to predict the type of bacteria from a blinded testing set of fluorescence data. The five oral bacteria were classified with 100% accuracy of discrimination, and the assay proved to provide a highly effective sensor array for pathogen identification, as shown in Fig. 7(d).

The increase in emission intensity indicates that the bacteria work as an electron donor, whereas the DAP NPs serves as an electron acceptor.^71^ The unique fluorescence response of each DAP NPs to each bacterium may be attributed to the fact that each one of the five DAP NPs is varied in terms of the density, nature, and orientation of the surface functional groups, which directly affect the charge carrier trapping and excitation relaxation pathways and thus their fluorescence signal when interacted with the bacterial surface.

In clinical settings, the samples will contain mixed bacterial specimen. Thus the sensor array should be able to identify the oral bacteria in a complex sample with a bacterial blend. To this end, eight representative blended samples consisting of four mixtures of two species of microbes and four mixtures of three species of microbes were used as the targeted mixed culture of the oral bacteria. The ML tool and the LDA-based tool were found to be very valuable for properly analyzing the photoluminescence data.^72^^–^^75^ LDA was used to transform the fluorescence output obtained from the sensor array as a response to bacterial mixtures to a 2D canonical score plot. The LDA algorithm was able to cluster the eight blended pathogens into eight groups with a 100% discrimination accuracy [Fig. 7(e)]. Based on the established function from the training part, 16 randomly selected blended samples were also completely identified in a blind test with a detection accuracy of 100%. As a practical application of the DAP NPs, it is proved that these particles can be used to construct a sensing array to detect bacteria. The combined use of this array along with a powerful algorithm can further expand the application of the array to identify the type of pathogen in a tested sample. We recognize that, under complex *in vivo* conditions, the specificity of CDs for binding to target bacterial species can be a limitation. An efficient computational algorithm will therefore play a vital role in this study to ensure that the CDs only bind to the desired bacteria and not to other non-target species that may be present in the oral environment.

Further, these experimental results were corroborated with computational studies. The differential response of DAP NPs toward different microbial strains led us to investigate their interaction with various surface proteins available on the bacteria. It may be hypothesized that, over the short exposure time of NPs to the bacterial strains, the CDs could only interact with the surface exposed proteins to generate a differential sensing response. Accordingly, we considered maltodextrin binding protein MalE1 for *L. casei* (PDB ID: 5 MTU); ${\mathrm{Zn}}^{2+}$-dependent intercellular adhesion protein for *S. epidermidis* (PDB ID: 4 FUM); V-region of antigen I/II for *S. mutans* (PDB ID: 1 JMM); type III-A Csm-CTR1 complex, AMPPNP bound protein of *S. salivarius* (PDB ID: 6 IFK) and SrpA Adhesin protein of *S. sanguinis* (PDB ID: 5EQ2) (Fig. S23 in the Supplementary Material).

**Fig. 8:**
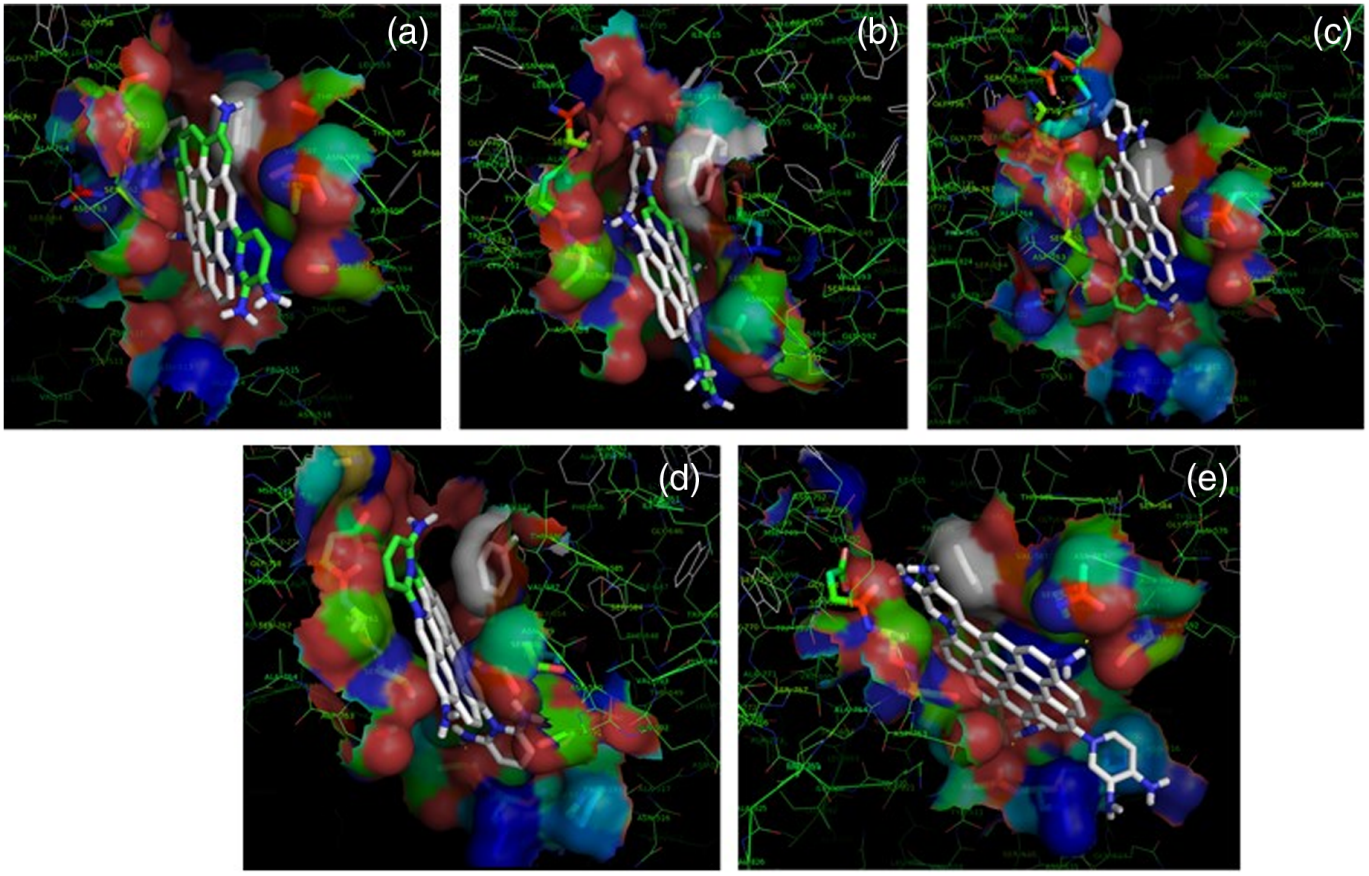
Representative pictorial representation of the most stable docked geometries of V-region of antigen I/II for *S. mutans* (PDB ID: 1JMM) with (a) 2,3 DAP NPs, (b) 2,4 DAP NPs, (c) 2,5 DAP NPs, (d) 2,6 DAP NPs, and (e) 3,4 DAP NPs.

On the other hand, the model structures of DAPCDs were built as different ovalene derivatives^48^ and energy minimized by the B3LYP/3-21G(d) method^76^ in GAMESS software.^51^ The HOMO–LUMO levels were also calculated; these indicated that HOMO was largely located over the graphene-like π-motif structures, whereas LUMO was located over the pyridine-like moieties for all of the structures (Fig. S24 in the Supplementary Material). The HOMO–LUMO gap was found to be the lowest for 2,5 DAP NPs, indicating its plausible faster response toward the analytes. These energy-minimized CDs were then docked against the surface exposed proteins of the studied bacteria, and the results were closely monitored. Table S2 in the Supplementary Material shows the comparative free energies of binding and clustering efficiencies of CDs with the surface proteins. Comparing both the average free energy of binding and clustering efficiency of the DAP derivatives, it can be concluded that 2,5 DAP NPs interacted more strongly with the surface proteins of *L. casei*, *S. salivarius*, and *S. sanguinis*, whereas 2,3 DAP NPs and 2,6 DAP NPs interacted more strongly with the surface proteins of *S. epidermidis* and *S. mutans*, respectively. It is to be noted that the trend might differ with the change in surface proteins under study. The most stable docked geometries of these protein–ligand complexes are presented in Fig. 8 and Fig. S25–S29 in the Supplementary Material, and the histograms representing the conformational binding energies of each of the complexes were shown in Tables S3–S7 in the Supplementary Material.

## 4 Conclusions

The hydrothermally derived CDs prepared under the same controlled condition from DAP isomers and urea were synthesized and characterized for their photophysical properties. We indicated via a comprehensive bulk state and single-particle level investigation that the PL properties of CDs can be regulated by the precursors’ isomeric position of nitrogen. We attributed these changes to the molecular products formed during the synthesis; their photophysical responses were predicted by DFT calculations. We emancipated the difference in the PL properties of CDs in a rapid method relying on ML algorithms to segregate the bacterial species leading to dental biofilm, which has huge implications for oral health. In the future, we will explore the efficacy of these CDs in differentiating various pathogenic bacteria in dental plaque developed under *in vivo* conditions. However, the major limitation to this study is the stability, poor QY of the CDs under *in vivo* conditions. Our future approaches will utilize various coating materials to improve the *in vivo* efficacy of the system. Further, the biocompatibility and immunogenicity of these CDs under *in vivo* conditions will be explored in a detailed manner. In our follow-on studies, the *in vivo* photoluminescence of the CDs will also be analyzed through ML algorithms to automatically identify the presence of pathogenic bacteria.
