# Supplementary material for: Ensemble and single-particle level fluorescent fine-tuning of carbon dots via positional changes of amines toward “supervised” oral microbiome sensing
Source: J Biomed Opt. 2023 Jul 6;28(8):082807. doi: 10.1117/1.JBO.28.8.082807 (PMC10324603; doi:10.1117/1.JBO.28.8.082807)

Ensemble and single-particle level fluorescent fine-tuning of carbon dots *via* positional changes of amines towards the 'supervised' oral microbiome sensing

Fatemeh Ostadhossein[a,b,c], *Parikshit Moitra*[d], *Maha Alafeef*[a,d], *Dinabandhu Sar*[a,b], *Shannon D'Souza*[a,b], *Lily F. Benig*[a,b], *Michael Nelappana*[a,b], *Xuedong Huang*[e], *Julio Soares*[f], *Kai Zhang*[g], *Dipanjan Pan**[a,b,c,d,i,j,k]

[a] Department of Bioengineering, University of Illinois at Urbana-Champaign, 611 West Park Street, Urbana, IL, USA

[b] Mills Breast Cancer Institute, Carle Foundation Hospital, Urbana, Illinois.

[c] Beckman Institute of Advanced Science and Technology, University of Illinois at Urbana-Champaign

[d] Department of Nuclear Engineering, The Pennsylvania State University, 3058 Research Drive, State College, Pennsylvania 16801, United States

[f] Department of Chemistry, Fudan University, Shanghai 200433, P. R. China

[g] Frederick Seitz Materials Research Laboratory, University of Illinois at Urbana-Champaign, Urbana, IL, 61801 USA

[h] Department of Biochemistry, School of Molecular and Cellular Biology, University of Illinois at Urbana-Champaign, Urbana, IL, 61801, USA

[i] Department of Materials Science and Engineering, The Pennsylvania State University, University Park, Pennsylvania 16801, USA

[j] The Materials Research Institute, Millennium Science Complex, Pollock Road, University Park, PA 16802 USA

[k] Huck Institutes of the Life Sciences, 101 Huck Life Sciences Building, University Park, PA 16802 USA

* Corresponding author: dipanjan@psu.edu

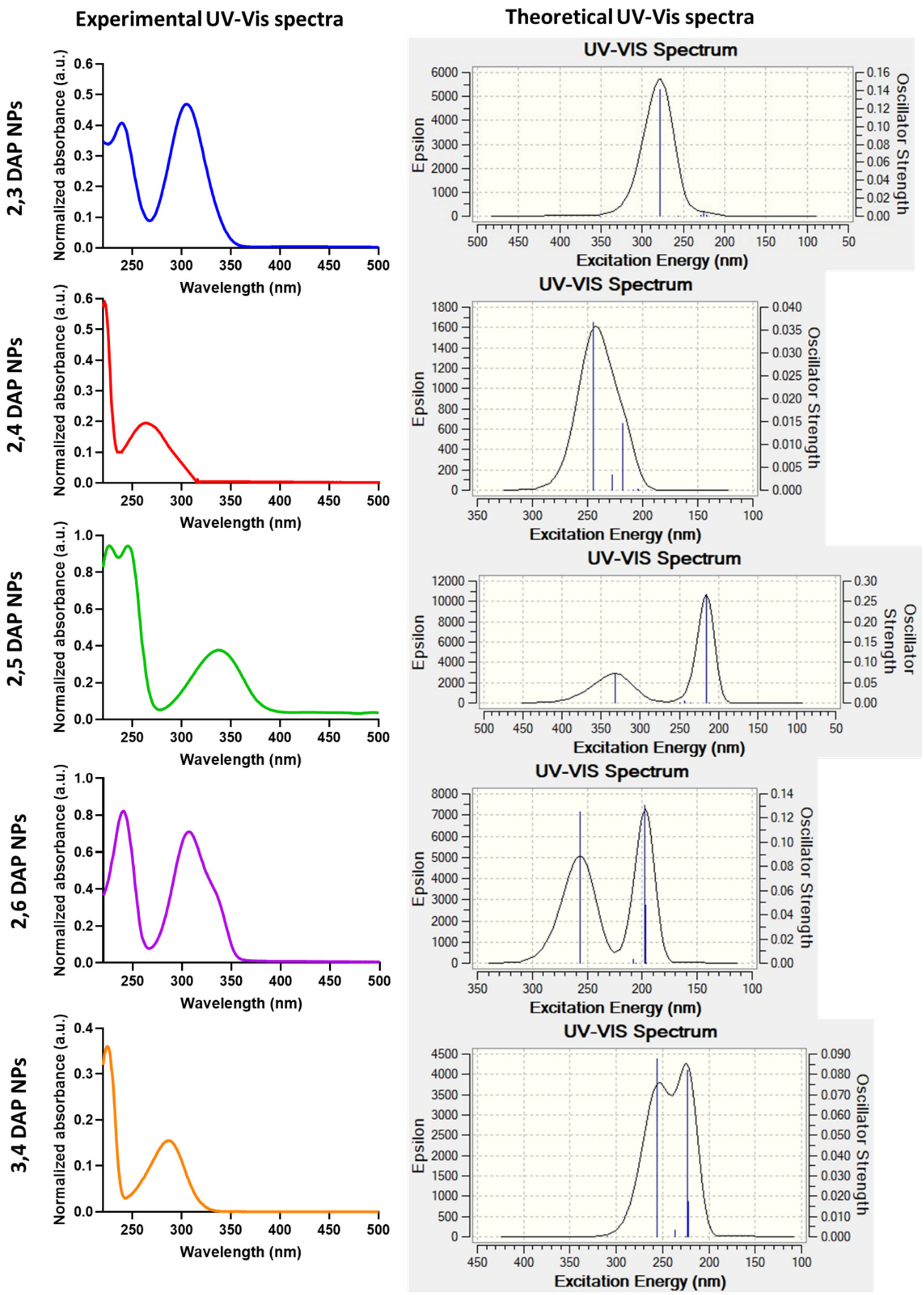

Fig. S1. Experimental and theoretical (from DFT) UV-Vis spectra for the NPs: from top to bottom: 2,3 DAP NPs; 2,4 DAP NPs; 2,5 DAP NPs; 2,6 DAP NPs and 3,4 DAP NPs.

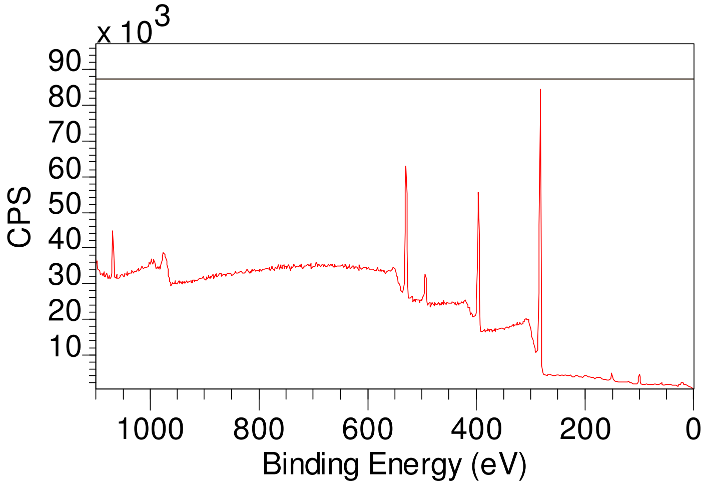

Fig. S2. XPS survey mode for 2,3 DAP NPs.

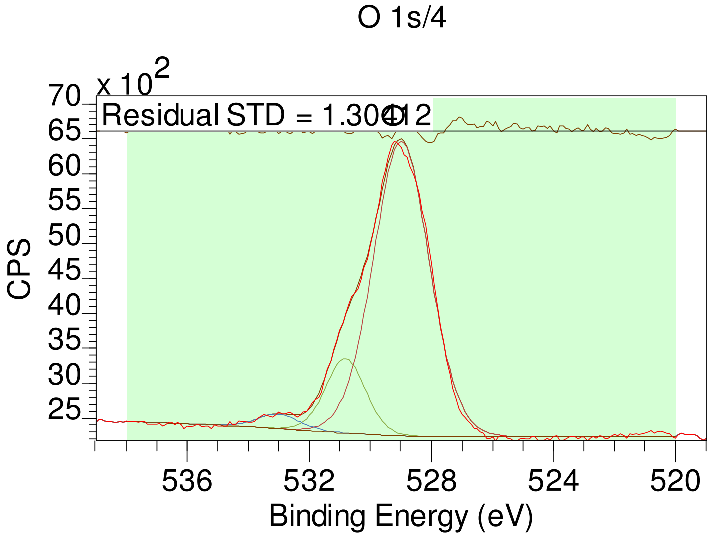

Fig. S3. XPS decomposition of O1S region for 2,3 DAP NPs.

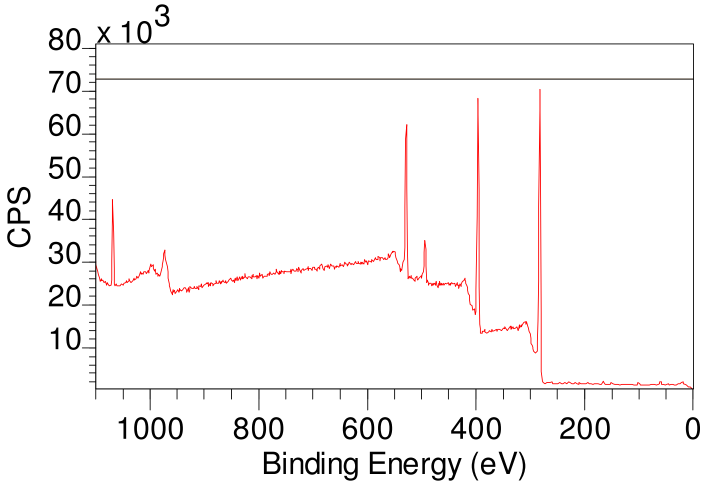

Fig. S4. XPS survey mode for 2,4 DAP NPs.

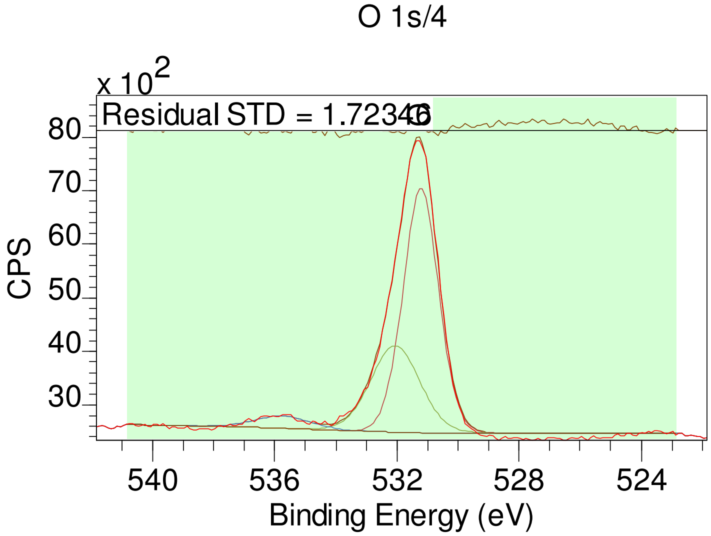

Fig. S5. XPS decomposition of O1S region for 2,4 DAP NPs.

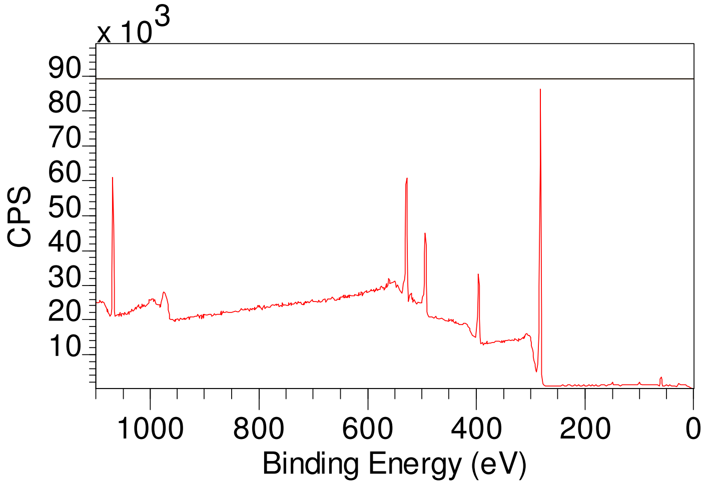

Fig. S6. XPS survey mode for 2,5 DAP NPs.

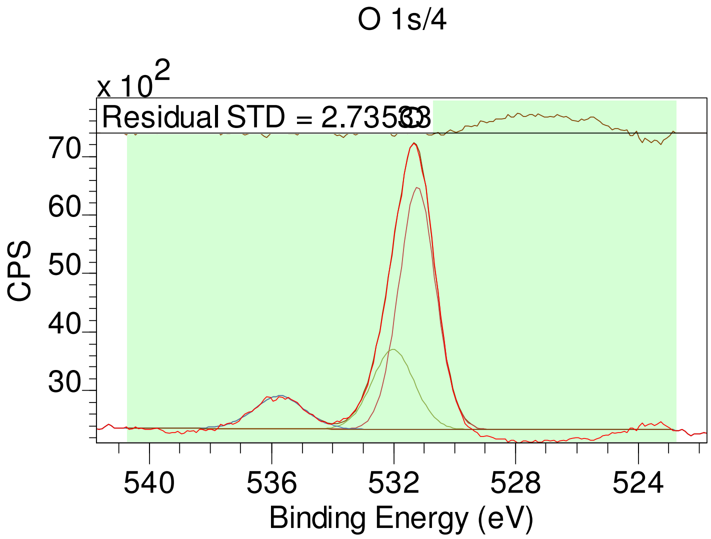

Fig. S7. XPS decomposition of O1S region for 2,6 DAP NPs.

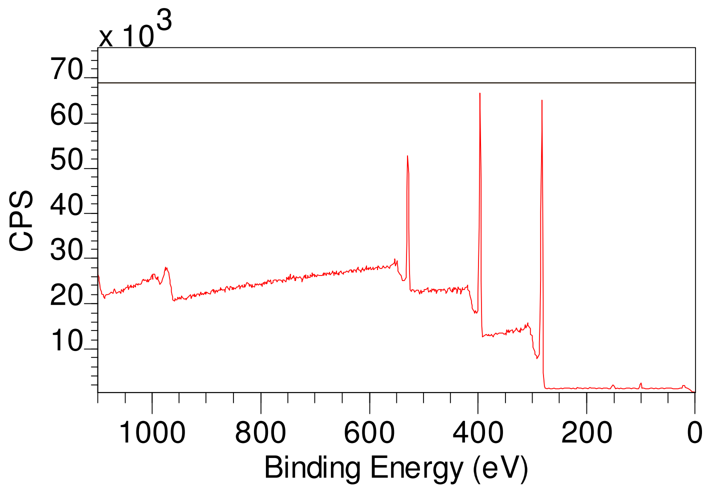

Fig. S8. XPS survey mode for 2,6 DAP NPs.

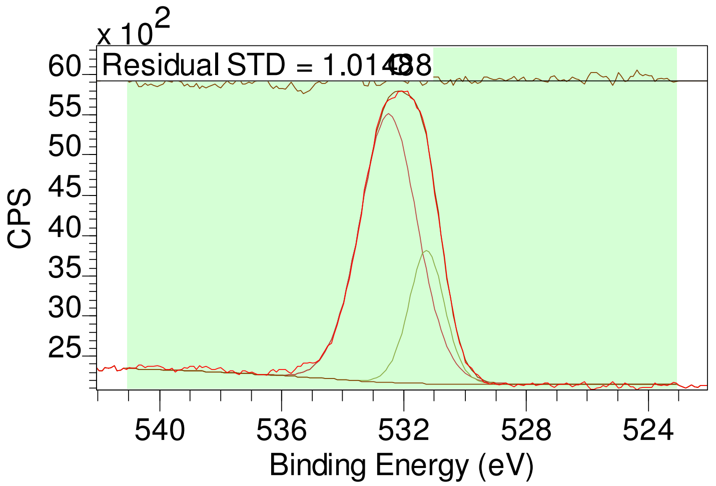

Fig. S9. XPS decomposition of O1S region for 2,6 DAP NPs.

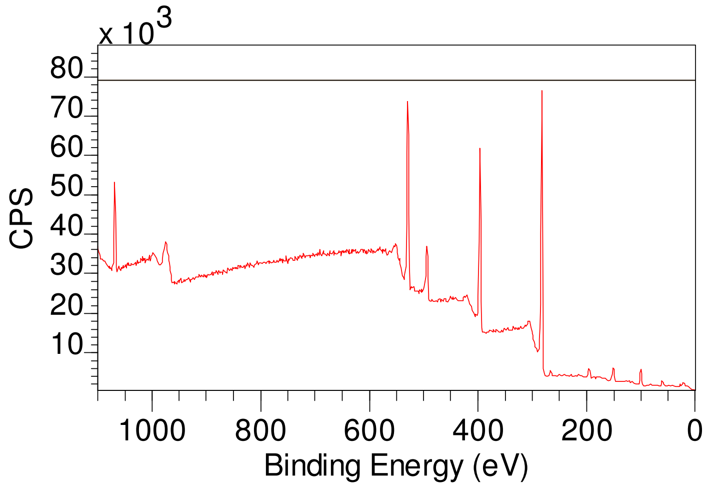

Fig. S10. XPS survey mode for 3,4 DAP NPs.

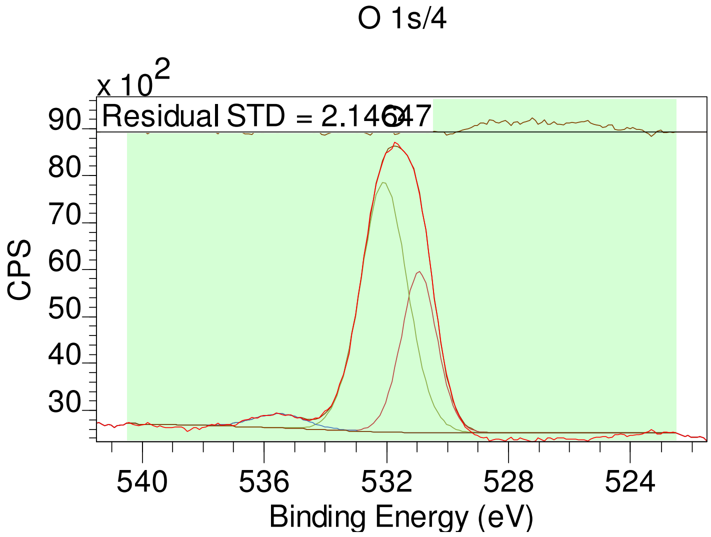

Fig. S11. XPS decomposition of O1S region for 2,3 DAP NPs.

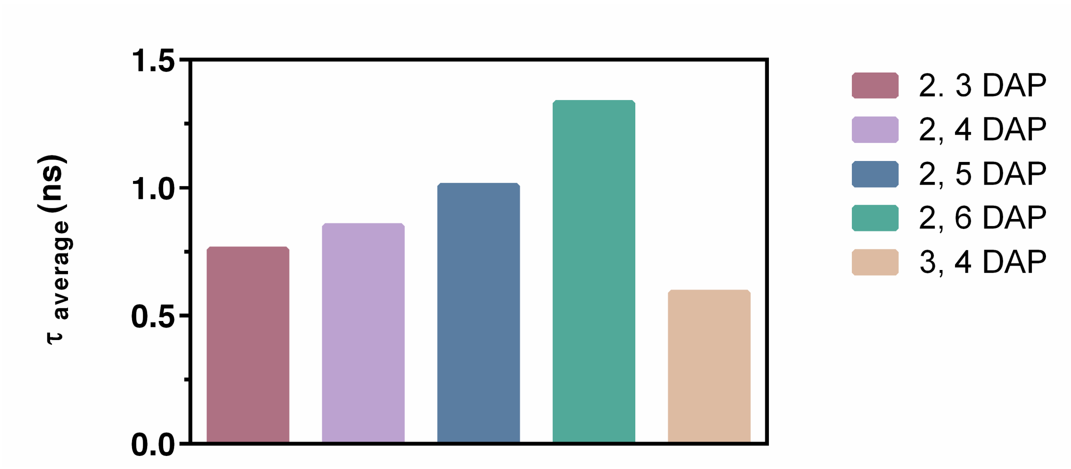

Fig. S12. TRPL average decay time for NPs when excited with 510 nm laser.

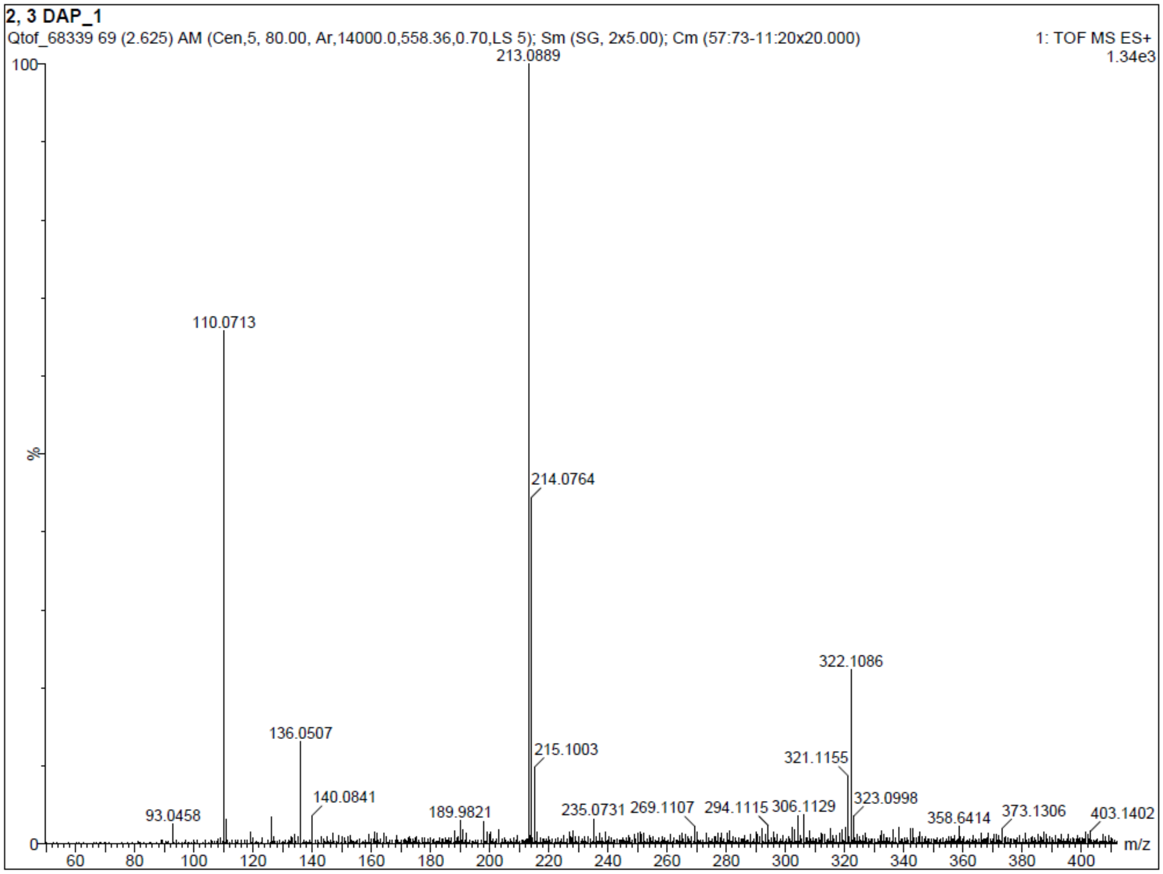

Fig. S13. Mass spec result of the 2,3 DAP NPs.

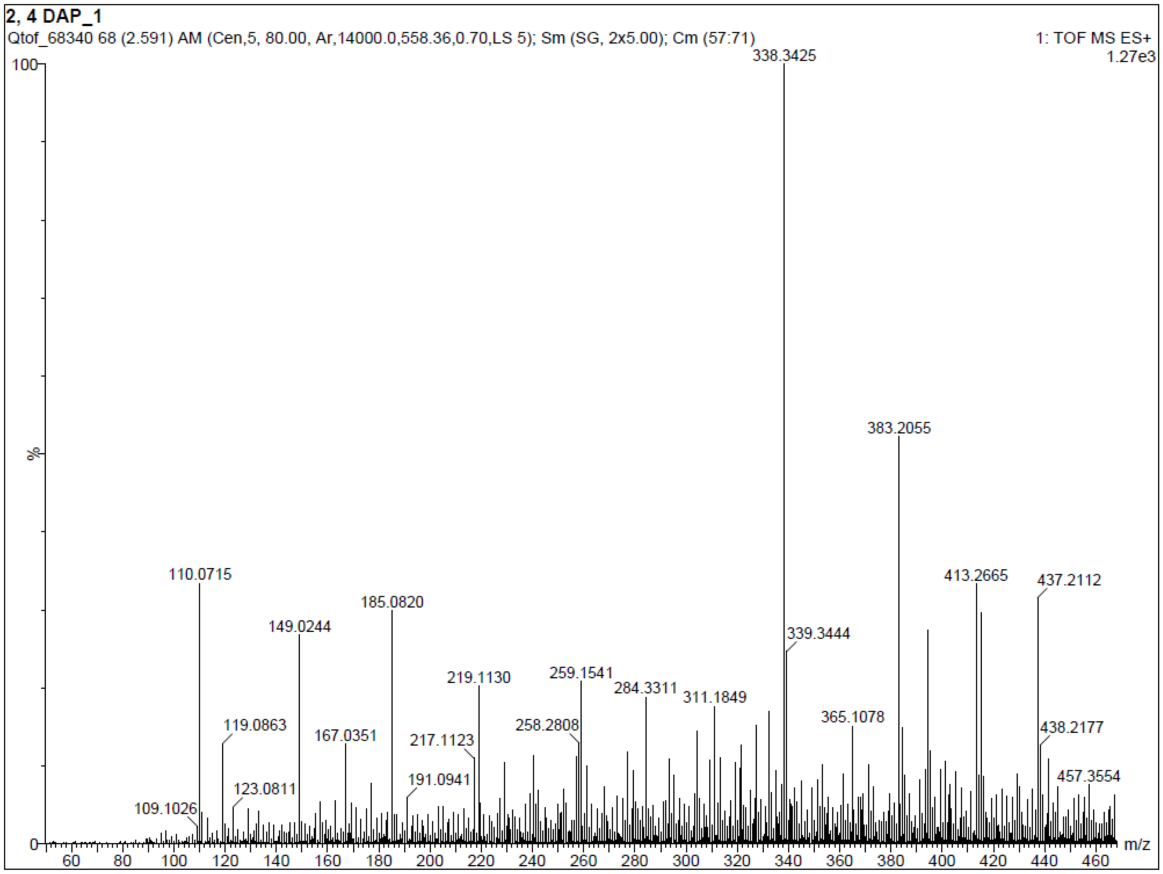

Fig. S14. Mass spec result of the 2,4 DAP NPs.

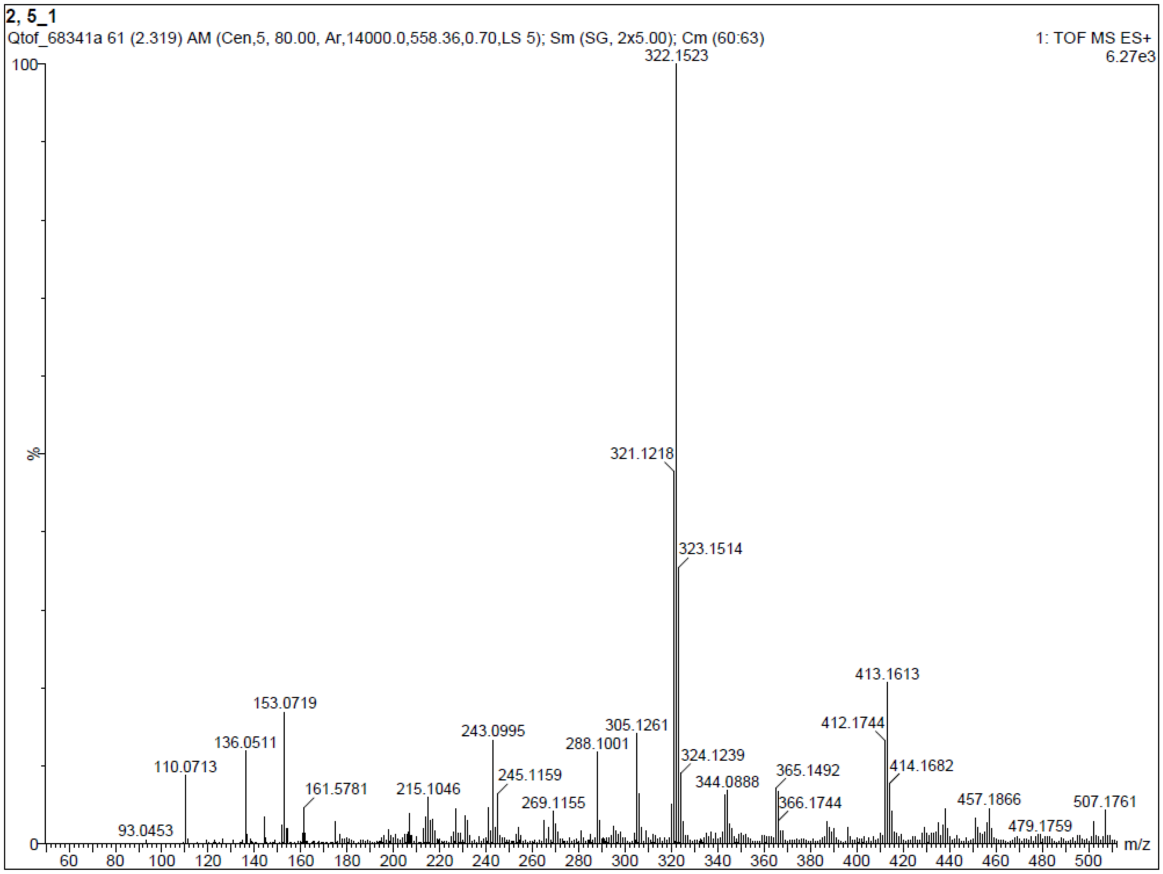

Fig. S15. Mass spec result of the 2,5 DAP NPs.

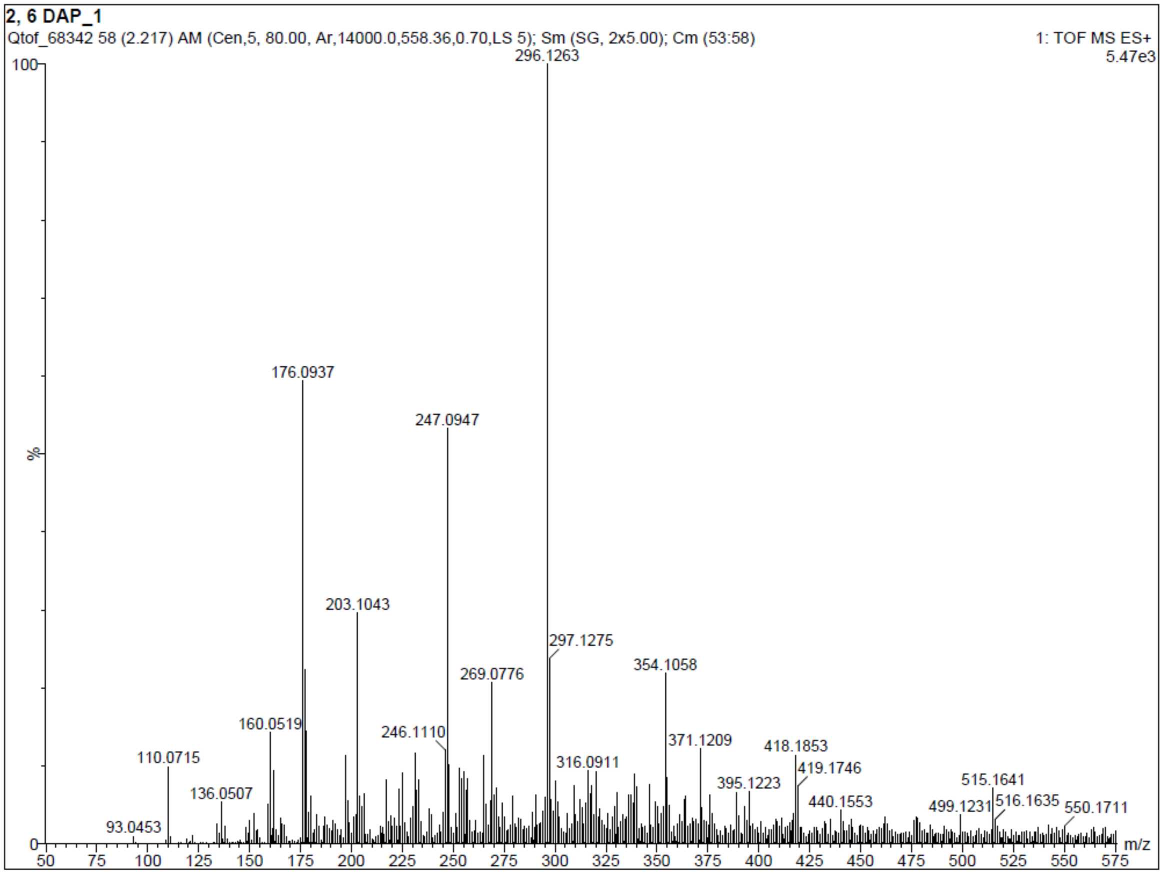

Fig. S16. Mass spec result of the 2,6 DAP NPs.

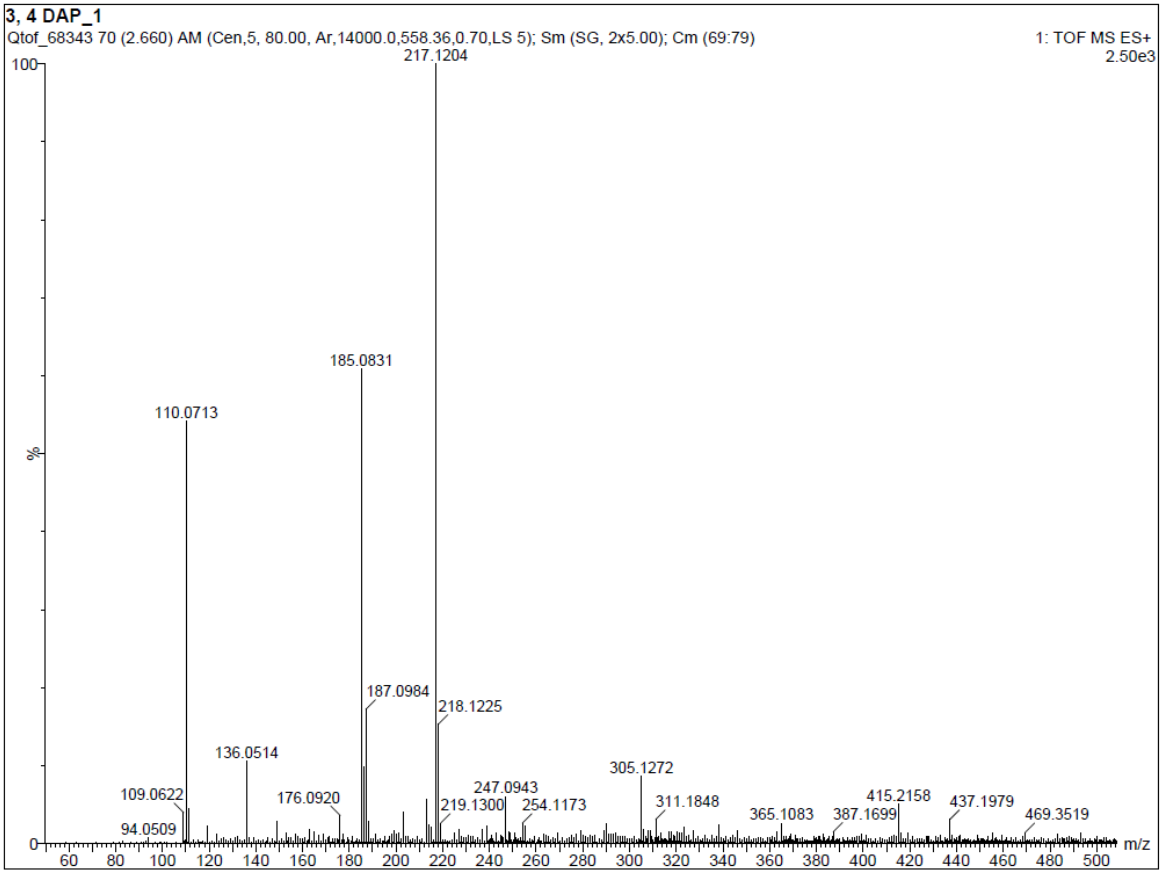

Fig. S17. Mass spec result of the 3,4 DAP NPs.

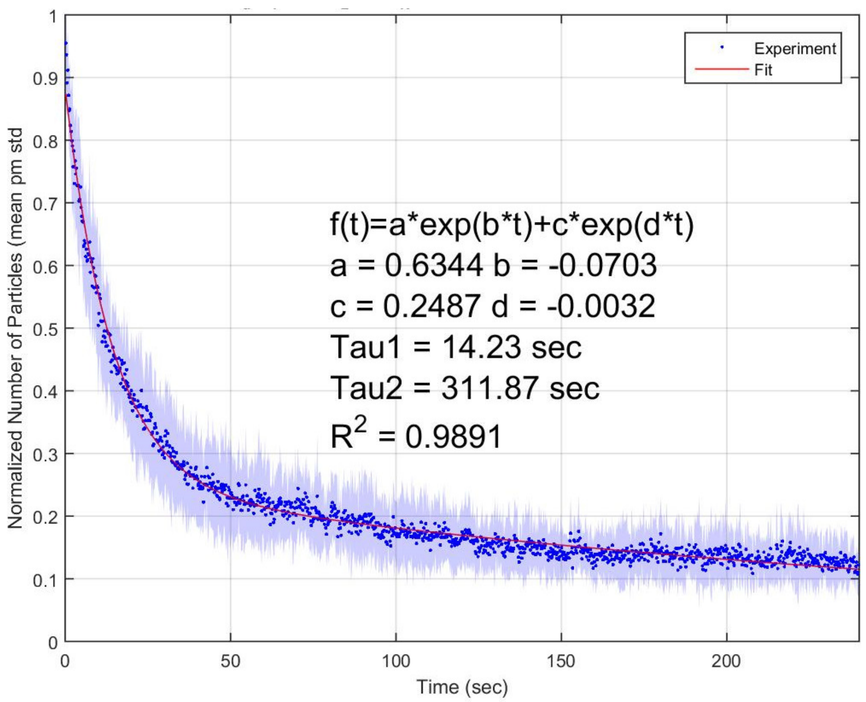

Fig. S18. The bleaching profile on the single particle level for 2,3 DAP NPs.

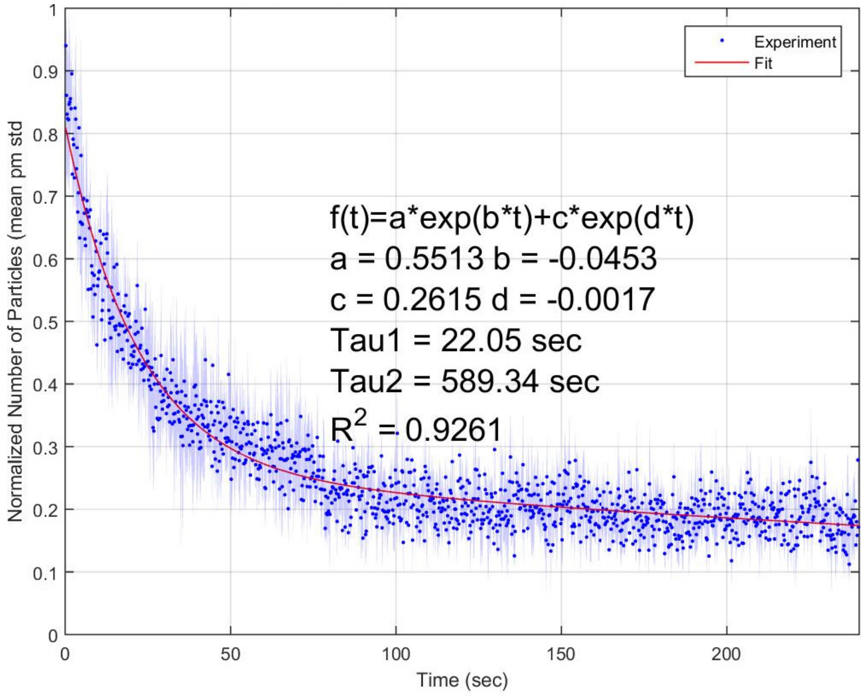

Fig. S19. The bleaching profile on the single particle level for 2,4 DAP NPs.

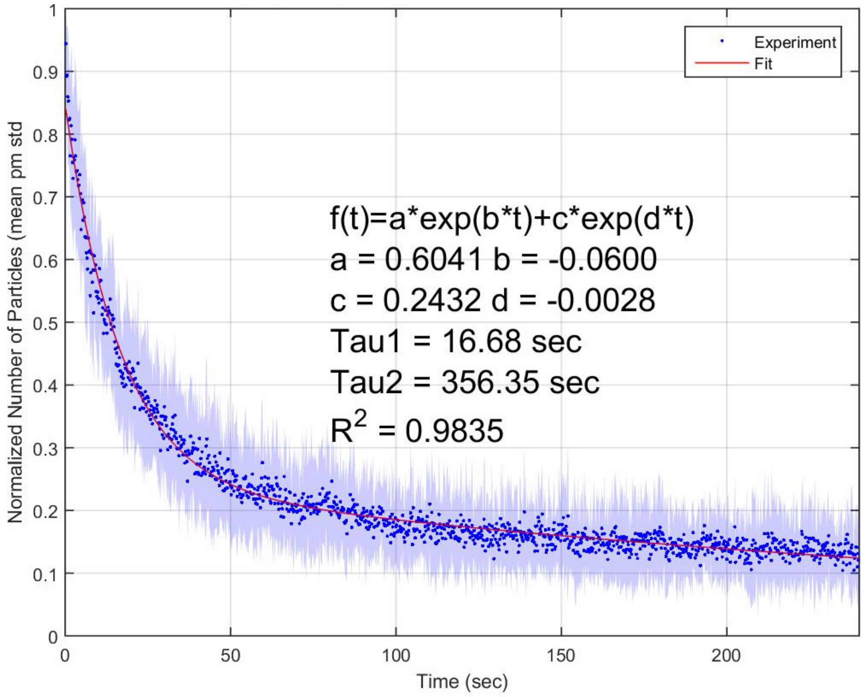

Fig. S20. The bleaching profile on the single particle level for 2,5 DAP NPs.

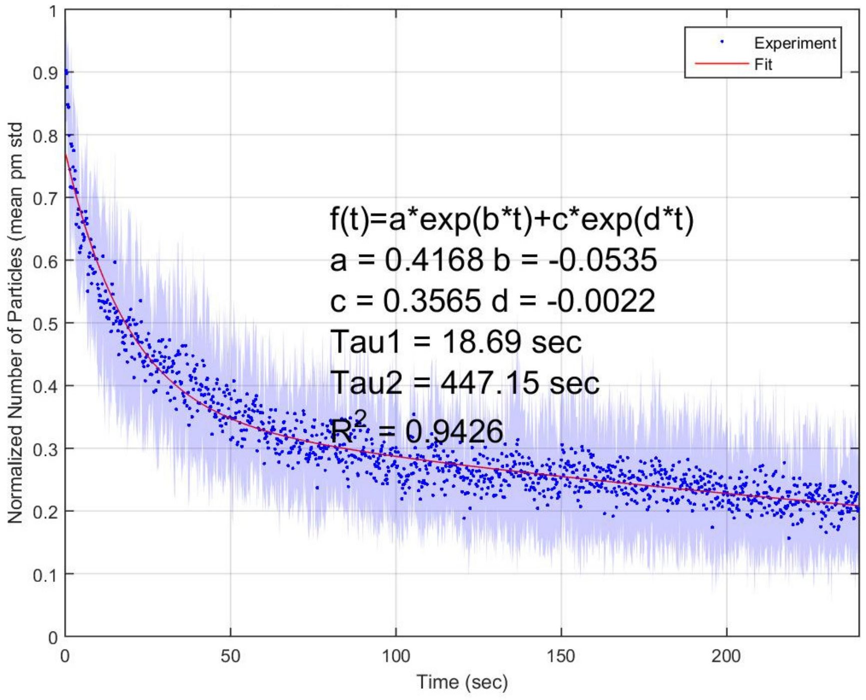

Fig. S21. The bleaching profile on the single particle level for 2,6 DAP NPs.

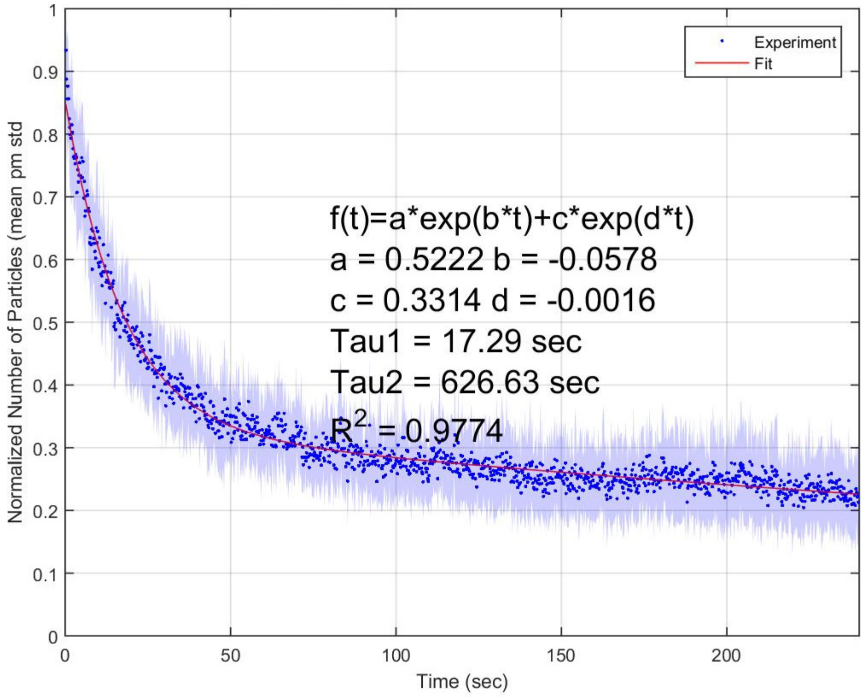

Fig. S22. The bleaching profile on the single particle level for 3,4 DAP NPs.

Table S1. Summary of the bleaching time

Sample	T1 (s)	T2 (s)
2,3 DAP NPs	14.2	311.9
2,5 DAP NPs	16.7	356.4
3,4 DAP NPs	17.3	626.6
2,6 DAP NPs	18.7	447.2
2,4 DAP NPs	22.0	589.3

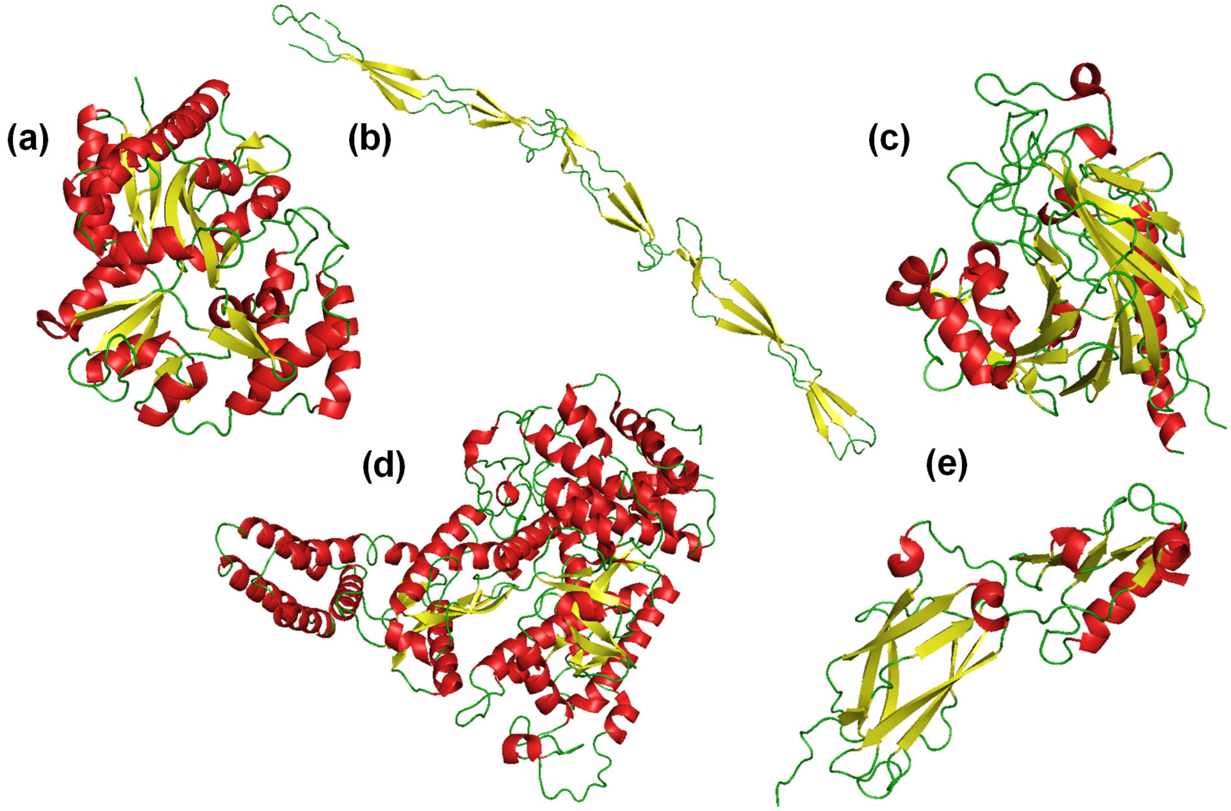

Fig. S23. PDB structures of (a) maltodextrin binding protein MalE1 for L. Casei (PDB ID: 5MTU); (b) Zn2+-dependent intercellular adhesion protein for S. epidermidis (PDB ID: 4FUM); (c) V-region of antigen I/II for S. mutans (PDB ID: 1JMM); (d) type III-A Csm-CTR1 complex, AMPPNP bound protein of S. salivarius (PDB ID: 6IFK) and (e) SrpA Adhesin protein of S. sanguinis (PDB ID: 5EQ2).

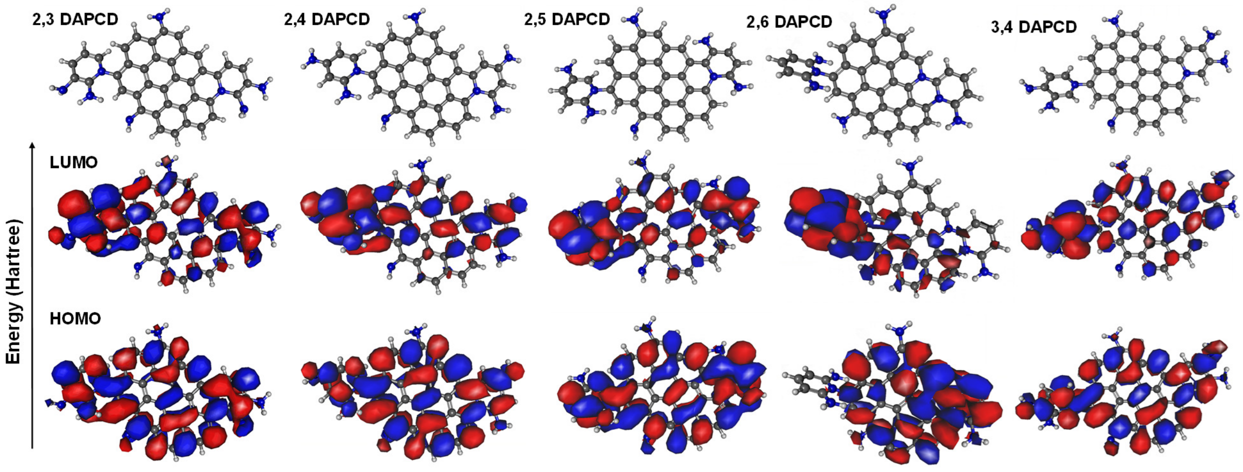

Fig. S24. Energy minimized structures of the DAPCD derivatives along with their HOMO-LUMO representations.

Table S2. Comparative free energies of binding and clustering efficiencies of DAPCD derivatives and various surface proteins of bacteria.

Protein	Compound	Average Free Energy of Binding (kcal/mol)	Clustering
L casei	2,3 DAPCD	-7.28	28/100
L casei	2,4 DAPCD	-7.20	29/100
L casei	2,5 DAPCD	-7.03	38/100
L casei	2,6 DAPCD	-7.56	25/100
L casei	3,4 DAPCD	-6.76	23/100
**Protein**	**Compound**	**Average Free Energy of Binding (kcal/mol)**	**Clustering**
S epidermidis	2,3 DAPCD	-6.90	46/100
S epidermidis	2,4 DAPCD	-6.80	29/100
S epidermidis	2,5 DAPCD	-6.86	33/100
S epidermidis	2,6 DAPCD	-6.94	27/100
S epidermidis	3,4 DAPCD	-6.51	33/100
**Protein**	**Compound**	**Average Free Energy of Binding (kcal/mol)**	**Clustering**
S mutans	2,3 DAPCD	-7.78	49/100
S mutans	2,4 DAPCD	-7.98	37/100
S mutans	2,5 DAPCD	-8.0	34/100
S mutans	2,6 DAPCD	-8.45	42/100
S mutans	3,4 DAPCD	-6.91	38/100
**Protein**	**Compound**	**Average Free Energy of Binding (kcal/mol)**	**Clustering**
S salivarius	2,3 DAPCD	-8.43	59/100
S salivarius	2,4 DAPCD	-8.97	42/100
S salivarius	2,5 DAPCD	-9.13	43/100
S salivarius	2,6 DAPCD	-8.07	38/100
S salivarius	3,4 DAPCD	-8.76	49/100

Protein	Compound	Average Free Energy of Binding (kcal/mol)	Clustering
S sanguinis	2,3 DAPCD	-7.93	44/100
S sanguinis	2,4 DAPCD	-8.20	38/100
S sanguinis	2,5 DAPCD	-8.14	39/100
S sanguinis	2,6 DAPCD	-7.61	26/100
S sanguinis	3,4 DAPCD	-7.46	38/100

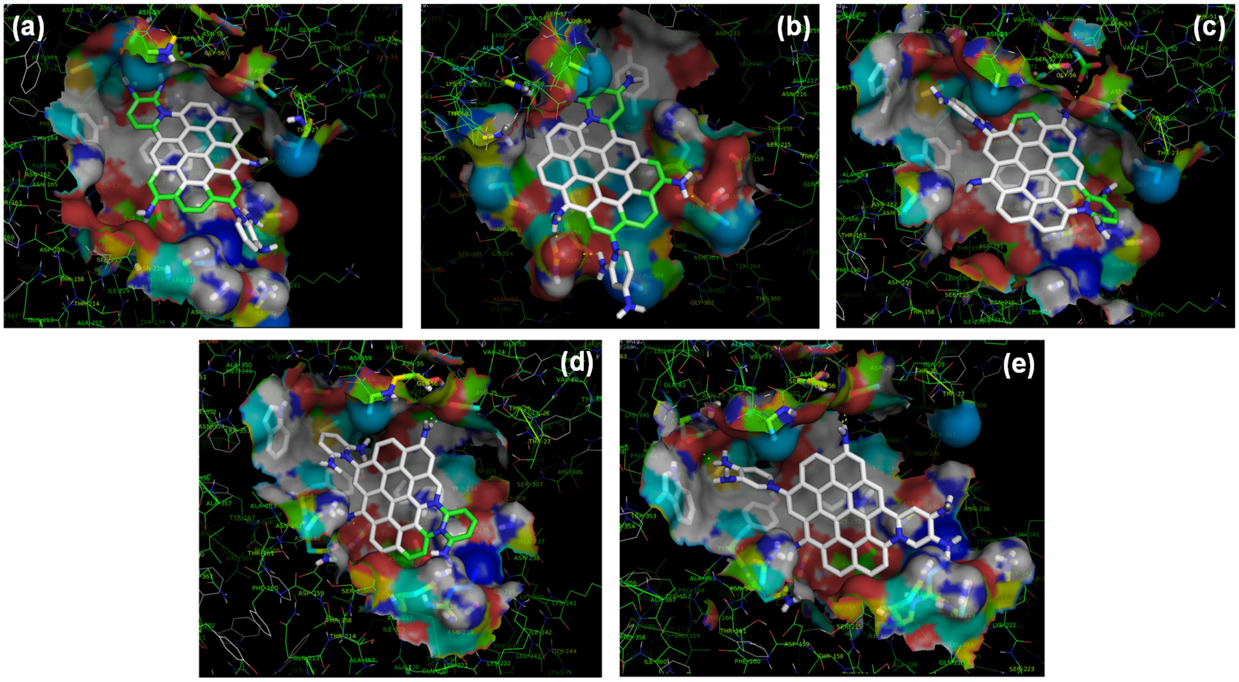

Fig. S25. Pictorial representation of the most stable docked geometries of maltodextrin binding protein MalE1 for L. Casei (PDB ID: 5MTU) with (a) 2,3, DAP NPs , (b) 2,4, DAP NPs , (c) 2,5, DAP NPs , (d) 2,6, DAP NPs and (e) 3,4, DAP NPs .

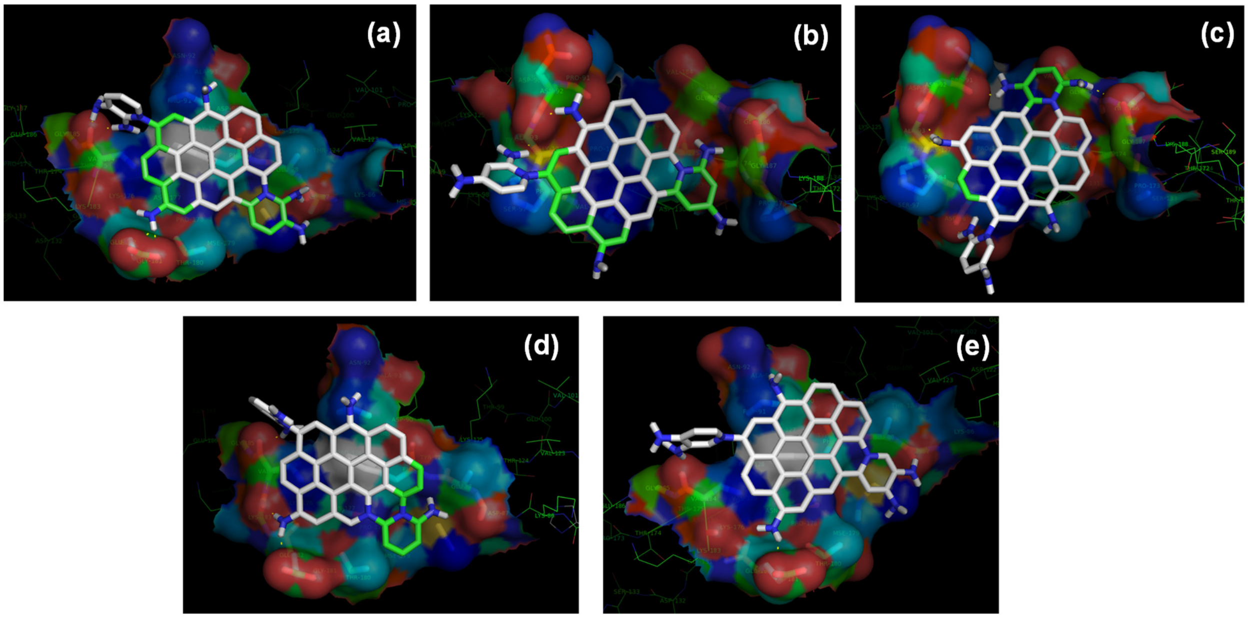

Fig. S26. Pictorial representation of the most stable docked geometries of Zn^{2+}-dependent intercellular adhesion protein for *S. epidermidis* (PDB ID: 4FUM) with (a) 2,3, DAP NPs , (b) 2,4, DAP NPs , (c) 2,5, DAP NPs , (d) 2,6, DAP NPs and (e) 3,4, DAP NPs .

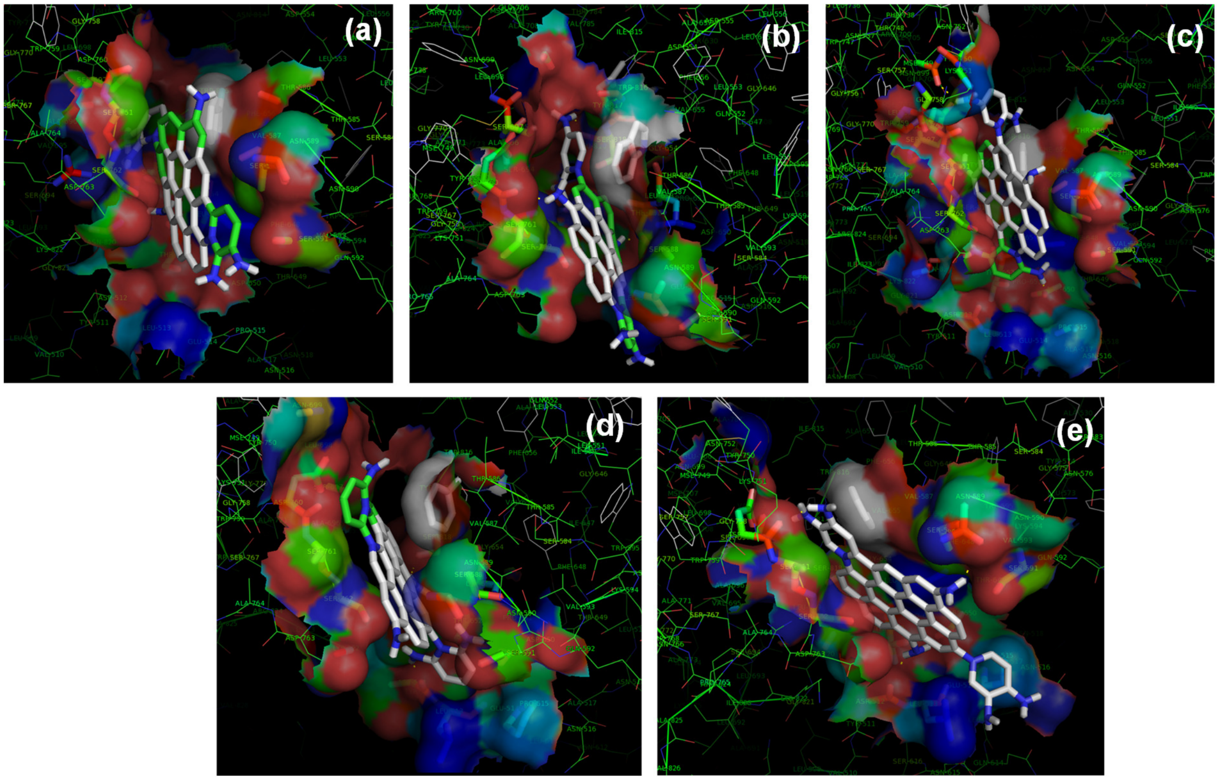

Fig. S27. Pictorial representation of the most stable docked geometries of V-region of antigen I/II for *S. mutans* (PDB ID: 1JMM) with (a) 2,3, DAP NPs , (b) 2,4, DAP NPs , (c) 2,5, DAP NPs , (d) 2,6, DAP NPs and (e) 3,4, DAP NPs .

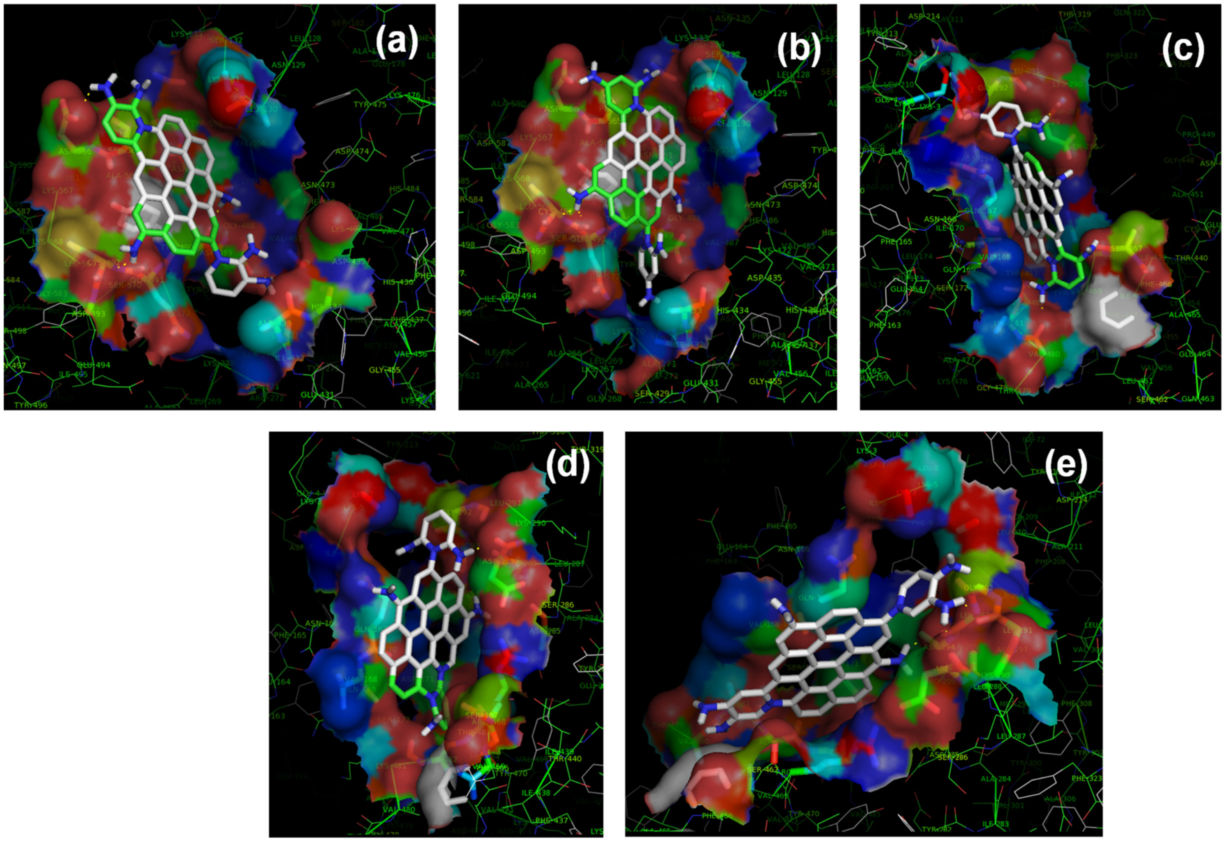

Fig. S28. Pictorial representation of the most stable docked geometries of type III-A Csm-CTR1 complex, AMPPNP bound protein of *S. salivarius* (PDB ID: 6IFK) with (a) 2,3, DAP NPs , (b) 2,4, DAP NPs , (c) 2,5, DAP NPs , (d) 2,6, DAP NPs and (e) 3,4, DAP NPs .

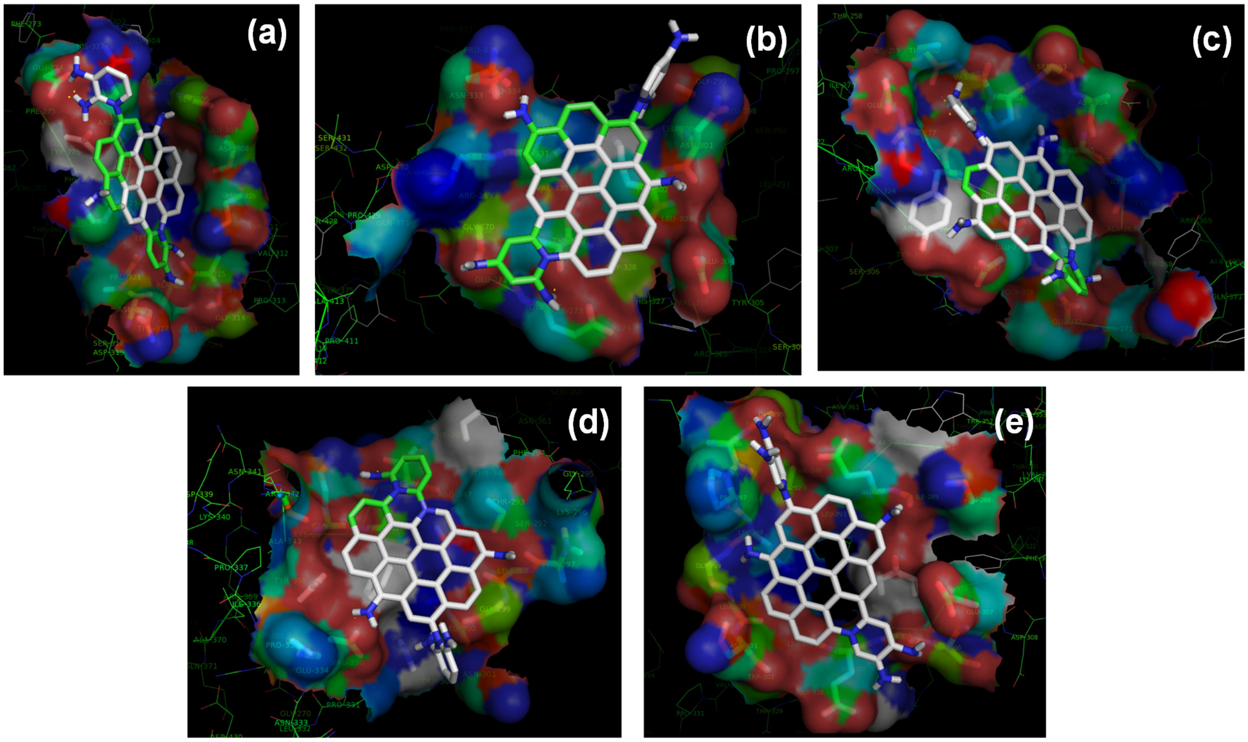

Fig. S29. Pictorial representation of the most stable docked geometries of SrpA Adhesin protein of *S. sanguinis* (PDB ID: 5EQ2) with (a) 2,3, DAP NPs , (b) 2,4, DAP NPs , (c) 2,5, DAP NPs , (d) 2,6, DAP NPs and (e) 3,4, DAP NPs .

Table S3. Histogram representing the number of conformations with the binding energy of maltodextrin binding protein MalE1 for L. Casei (PDB ID: 5MTU) with (a) 2,3, DAP NPs , (b) 2,4, DAP NPs , (c) 2,5, DAP NPs , (d) 2,6, DAP NPs and (e) 3,4, DAP NPs .

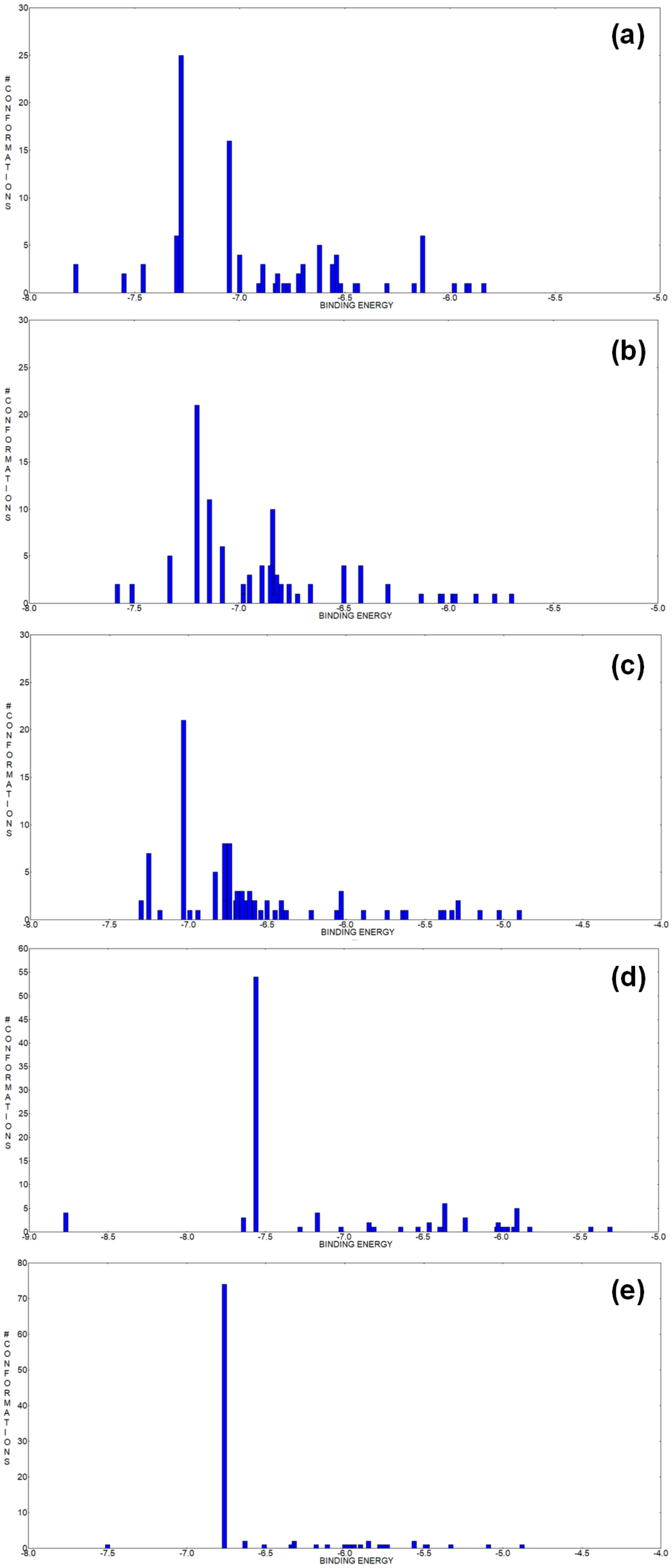

Table S4. Histogram representing the number of conformations with the binding energy of Zn2+-dependent intercellular adhesion protein for S. epidermidis (PDB ID: 4FUM) with (a) 2,3, DAP NPs , (b) 2,4, DAP NPs , (c) 2,5, DAP NPs , (d) 2,6, DAP NPs and (e) 3,4, DAP NPs .

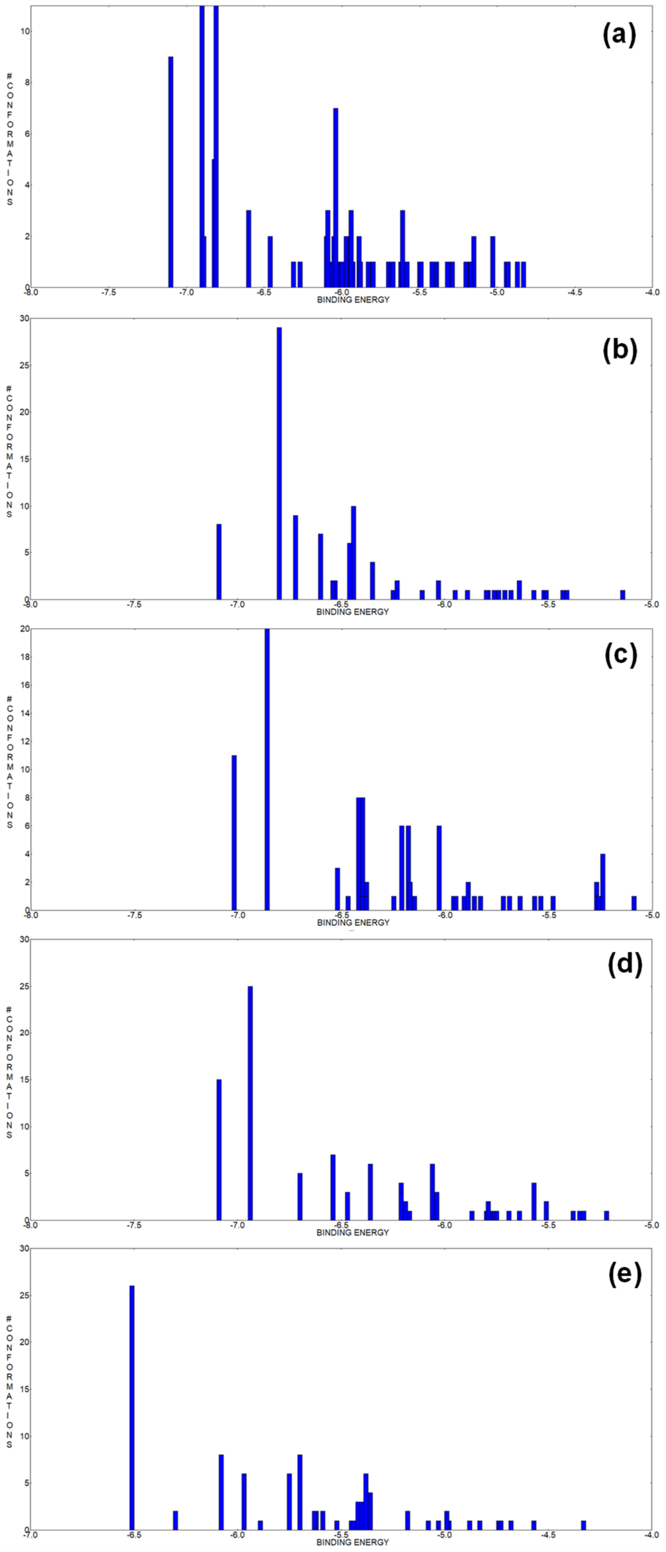

Table S5. Histogram representing the number of conformations with the binding energy of V-region of antigen I/II for S. mutans (PDB ID: 1JMM) with (a) 2,3, DAP NPs , (b) 2,4, DAP NPs , (c) 2,5, DAP NPs , (d) 2,6, DAP NPs and (e) 3,4, DAP NPs .

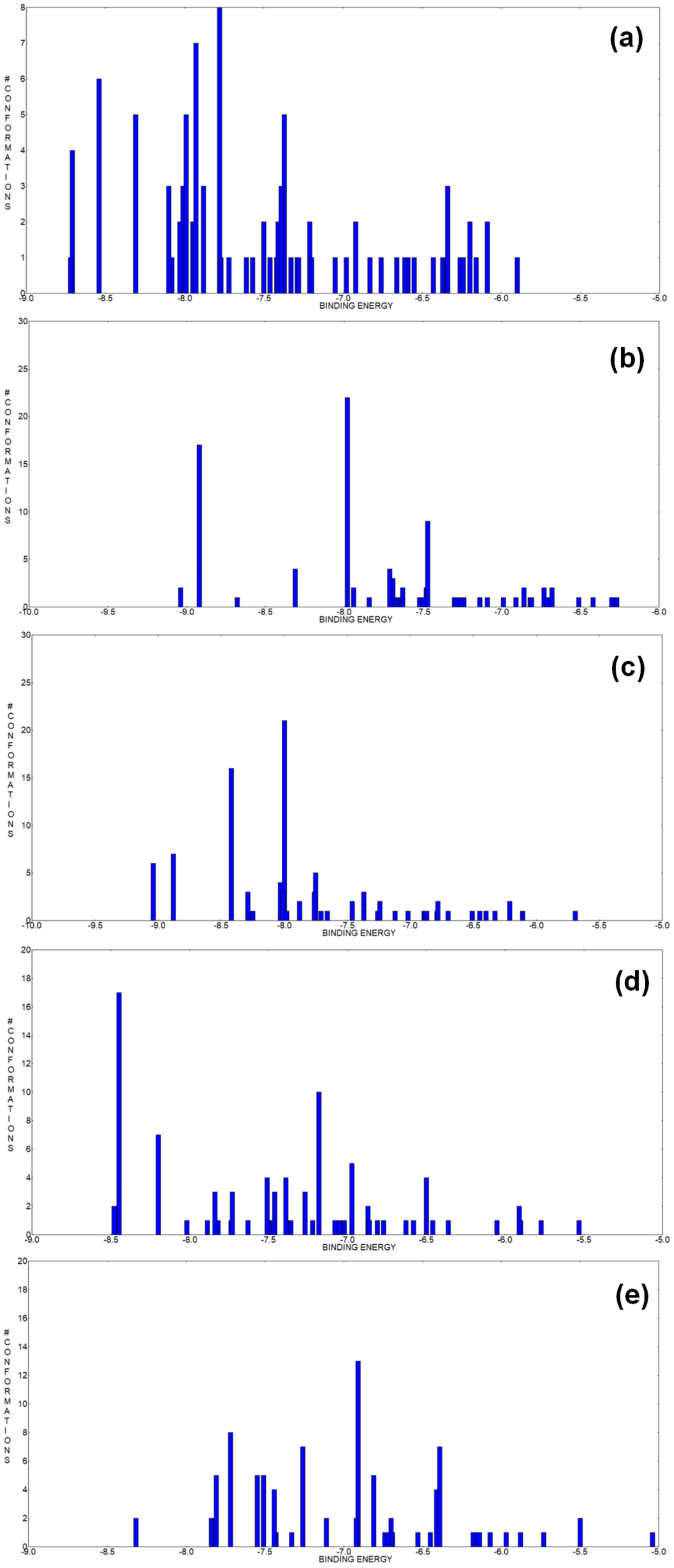

Table S6. Histogram representing the number of conformations with the binding energy of type III-A Csm-CTR1 complex, AMPPNP bound protein of S. salivarius (PDB ID: 6IFK) with (a) 2,3, DAP NPs , (b) 2,4, DAP NPs , (c) 2,5, DAP NPs , (d) 2,6, DAP NPs and (e) 3,4, DAP NPs .

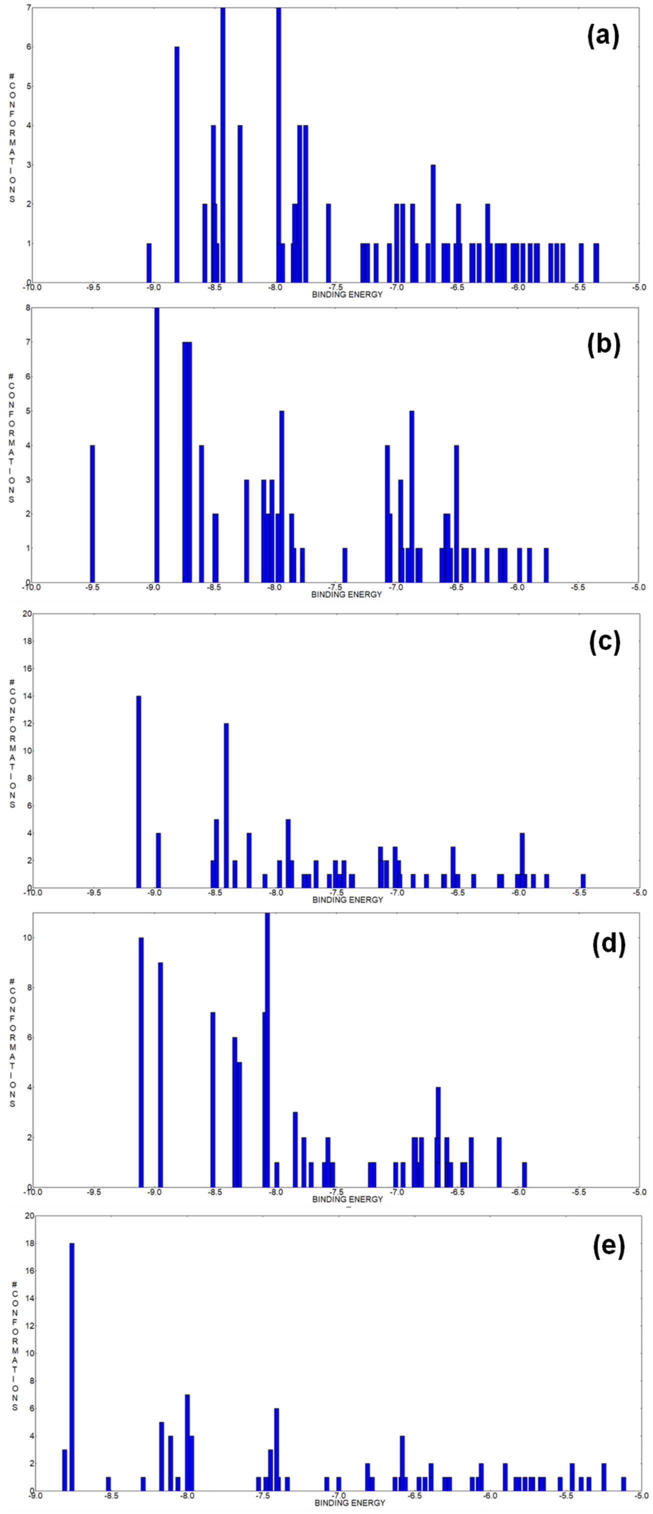

Table S7. Histogram representing the number of conformations with the binding energy of SrpA Adhesin protein of S. sanguinis (PDB ID: 5EQ2) with (a) 2,3, DAP NPs , (b) 2,4, DAP NPs , (c) 2,5, DAP NPs , (d) 2,6, DAP NPs and (e) 3,4, DAP NPs .

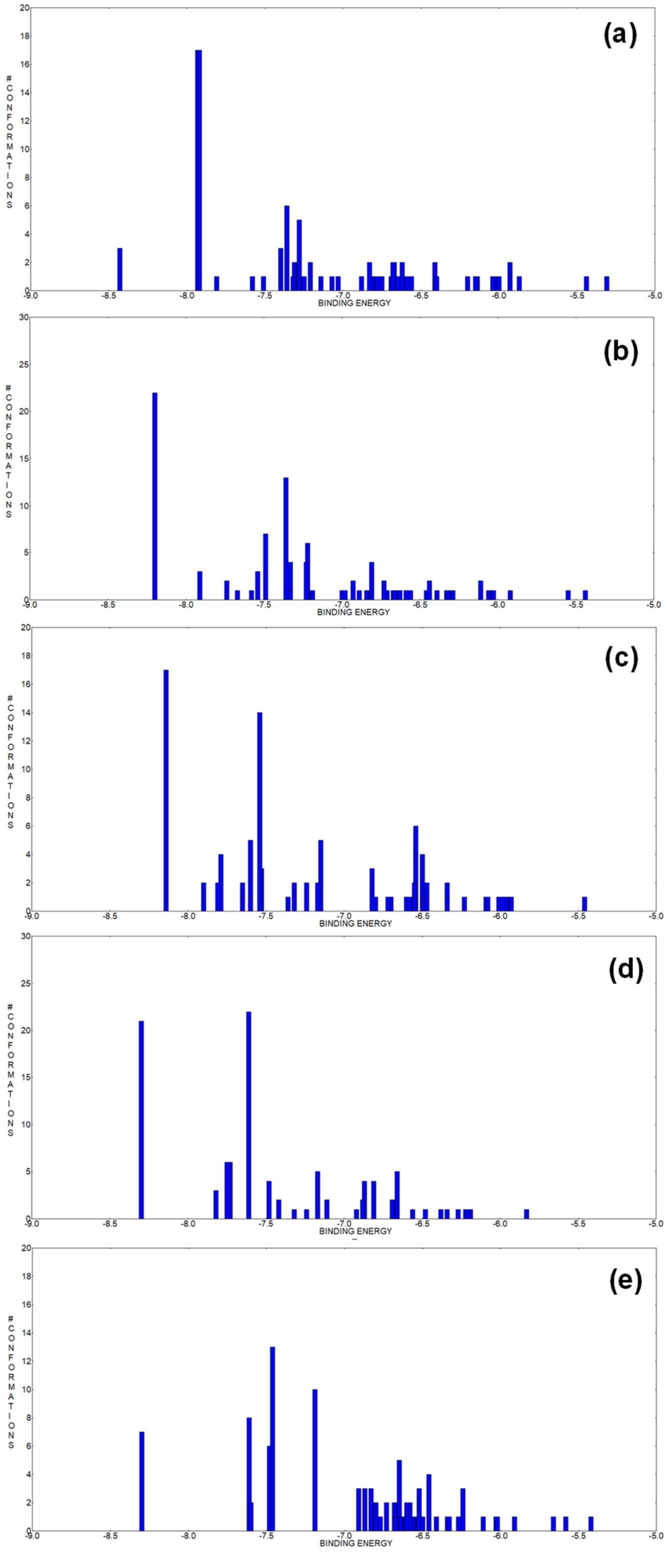

Supplement: Supplementary file 1 [file JBO_028_082807_SD001.pdf]
